# Computational methods for *in situ* structural studies with cryogenic electron tomography

**DOI:** 10.3389/fcimb.2023.1135013

**Published:** 2023-10-04

**Authors:** Cuicui Zhao, Da Lu, Qian Zhao, Chongjiao Ren, Huangtao Zhang, Jiaqi Zhai, Jiaxin Gou, Shilin Zhu, Yaqi Zhang, Xinqi Gong

**Affiliations:** ^1^ Mathematical Intelligence Application LAB, Institute for Mathematical Sciences, Renmin University of China, Beijing, China; ^2^ Beijing Academy of Intelligence, Beijing, China

**Keywords:** cryo-electron tomography (cryo-ET), subtomogram averaging (STA), microorganism *in situ*, 3D reconstruction, deep learning, mathematical models

## Abstract

Cryo-electron tomography (cryo-ET) plays a critical role in imaging microorganisms *in situ* in terms of further analyzing the working mechanisms of viruses and drug exploitation, among others. A data processing workflow for cryo-ET has been developed to reconstruct three-dimensional density maps and further build atomic models from a tilt series of two-dimensional projections. Low signal-to-noise ratio (SNR) and missing wedge are two major factors that make the reconstruction procedure challenging. Because only few near-atomic resolution structures have been reconstructed in cryo-ET, there is still much room to design new approaches to improve universal reconstruction resolutions. This review summarizes classical mathematical models and deep learning methods among general reconstruction steps. Moreover, we also discuss current limitations and prospects. This review can provide software and methods for each step of the entire procedure from tilt series by cryo-ET to 3D atomic structures. In addition, it can also help more experts in various fields comprehend a recent research trend in cryo-ET. Furthermore, we hope that more researchers can collaborate in developing computational methods and mathematical models for high-resolution three-dimensional structures from cryo-ET datasets.

## 1 Introduction

Cryo-electron tomography (cryo-ET) is an important imaging technique that provides high-resolution three-dimensional (3D) structures for biological specimens *in situ*. Observing the structure of biological specimens *in situ* can reflect the location of spatial interaction, and further help to analyze the mechanism and function of macromolecules. Single-particle analysis (SPA) in cryogenic electron microscopy (cryo-EM) is a mature approach to obtaining high-resolution 3D reconstructed structures, even imaging nearly native biomolecules in cells or organelles *in situ*. Indeed, the *in situ* single-particle analysis (isSPA) (Cheng et al., 2021) method is a potential competitor of cryo-ET. However, a complex system of biological macromolecules is still challenging to construct. Cryo-ET can play a key bridging role in forming high-resolution cellular landscapes *in situ* among multiple scales. Cryo-ET has been increasingly favored by structural biologists in the past few years. There are several advantages of cryo-ET. Firstly, some artificial alterations or modifications are averted in sample preparation, like particle isolation and purification. The native state can be cryo-preserved by vitrification (Plitzko and Baumeister, 2019). Secondly, cryo-ET allows the sample thickness of biological specimens below 500 nm (Khanna and Villa, 2022; Zuber and Lučić, 2022), such as viruses and bacterial cells. However, a thinner sample means higher resolution recorded in some sense. Thirdly, cryo-ET can analyze heterogeneous (Xu et al., 2021) and pleomorphic (Borgnia et al., 2008; Obr and Schur, 2019) structures like cells, viruses, and tissues (Borgnia et al., 2008). Different from cryo-EM, cryo-ET does not require many copies of the same specimen. Here, we focus on cryo-ET in microorganisms.

The high-resolution structures of microorganisms in cryo-ET can provide reliable 3D structures for drug development, target discovery, vaccine research, and so on. They can promote the development of science and technology. There are some actual examples of microorganisms using cryo-ET, like *Bdellovibrio bacteriovorus* (Borgnia et al., 2008), *Mycoplasma genitalium* (Seybert et al., 2018), *Candida glabrata* (Jiménez-Ortigosa et al., 2021), *Mycoplasma pneumoniae* (Xue et al., 2022), and *Escherichia coli* (Chen et al., 2022). Recently, cryo-ET has resolved a giant virus (Fang et al., 2019) at the near-atomic level, namely, a 3.5-Å paramecium bursaria chlorella virus 1 (PBCV-1). The reorganization and host–pathogen interaction (Nans et al., 2014; López-Jiménez and Mostowy, 2021) are also resolved in cryo-ET. The structures further help to analyze the action mechanism of infected cells, which helps researchers to discover anti-infective strategies. Some viruses are also resolved in cryo-ET like severe acute respiratory syndrome coronavirus 2 (SARS-CoV-2) (Klein et al., 2020), human immunodeficiency virus (HIV) (Mendonҫa et al., 2021), and a bullet-shaped vesicular stomatitis virus (Jenni et al., 2022). As coronavirus disease 2019 (COVID-19) has caused great damage and loss to humans, the analysis of SARS-CoV-2 has become an important application of cryo-ET. The 3D structures of SARS-CoV-2 (Turoňová et al., 2020; Calder et al., 2022) by cryo-ET have helped researchers to explore the functional mechanism and promote the development of vaccines and drugs. Critical SARS-CoV-2 structures are resolved in Zhang et al. (2021), such as the virus egress pathway and native virus spike structures. The spike protein plays an important role in virus–host interaction, membrane fusion, and so on. The spike protein is a main target for vaccine development and antigenic analysis (Li, 2022). The structural analysis for alphacoronavirus spike protein *in situ* revealed the motion of D0 domain (Huang et al., 2022). However, there are still a large number of structures that have not been resolved. It is significant and urgent to develop methods for each step of reconstruction processing from tilt series in cryo-ET to restore high-resolution structures.

There are two main limiting factors for 3D structure reconstruction steps. On the one hand, a signal-to-noise ratio (SNR) (Bepler et al., 2020) is the ratio of signal power to noise power, which is often expressed in decibels (dB). Higher SNR means that the signal is stronger and the noise is weaker; that is, the quality of the image is better. On the other hand, the tilt angles are generally from −70° to 70° in cryo-ET. Thus, the missing angles lead to a lack of information in two continuous, symmetrical wedge-shaped areas in Fourier space (Liu et al., 2022b), which is referred to the missing wedge of data. For convenience, we use “missing wedge” to mean “missing wedge of data” in the rest of the paper. Low SNR and missing wedge are two factors that make the reconstruction procedure difficult. In addition, inter-structure heterogeneity can also negatively affect the reconstruction procedure. Structural heterogeneity refers to the fact that macromolecules have many different discrete or continuous conformations. Heterogeneity can affect the steps of 3D particle picking, classification of 3D particles, and so on.

**Figure 1:**
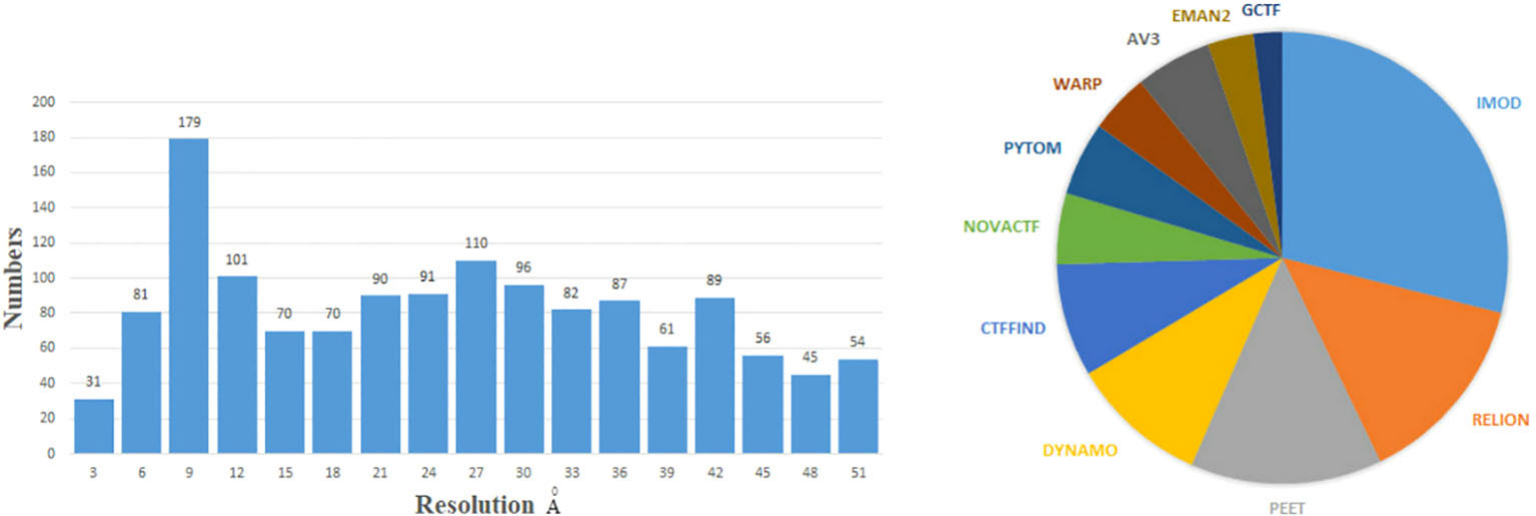
Left: the numbers of 3D density maps as a function of resolutions by the subtomogram averaging (STA) in cryo-ET. Here, we only capture numbers of the resolution from 1 to 51 Å. Right: the numbers of times the software is used by the STA. Specific data are before 28 December 2022 from the EMDB website: https://www.ebi.ac.uk/emdb/.

Data websites and software for cryo-ET have been developed in the past few years. The Electron Microscopy Data Bank (EMDB) (Lawson et al., 2016) and Electron Microscopy Public Image Archive (EMPIAR) (Iudin et al., 2016) are two popular databases. The EMPIAR provides tilt series projections, and EMDB provides 3D density maps. Subtomogram averaging (STA) is a technique of averaging 3D biological samples within tomograms (Bharat and Scheres, 2016b). The STA is a popular image restoration technique in cryo-ET to obtain the 3D structure of biological samples in their native environments (Scaramuzza and Castaño-Díez, 2021). The STA technique is increasingly important in structural biology. The concepts of each reconstruction steps will be described in specific sections. To demonstrate the current ability of cryo-ET to resolve structures, we show the resolution distribution and software rank by the STA from the EMDB before 28 December 2022, as **Figure 1** shows. The resolution of most biological samples analyzed by cryo-ET is low, and only a tiny part can reach more than 10 Å resolution, which is far from enough for the study of the internal mechanism of organisms. The top 11 software/methods are IMOD (Mastronarde and Held, 2017), Relion (Zivanov et al., 2022), Particle Estimation for Electron Tomography (PEET) (Nicastro et al., 2006), Dynamo (Scaramuzza and Castaño-Díez, 2021), Ctffind (Rohou and Grigorieff, 2015), Novactf (Turonova et al., 2017), PyTom (Hrabe et al., 2012), Warp (Tegunov and Cramer, 2019), Av3 (Förster et al., 2005), EMAN2 (Murray et al., 2014), and GCTF (Zhang, 2016). The ScipionTomo (Morena et al., 2022) provides a platform containing most software for users applying different software to solve the same task. Recently, with the rapid popularity of artificial intelligence algorithms, many new computational methods have emerged to improve the reconstruction resolution in cryo-ET. This review summarizes some new computational methods and classical mathematical models for 3D structure reconstruction from cryo-ET data. We hope that this review can attract more researchers to collaborate in developing reconstruction methods in cryo-ET and provide guidelines for other researchers.

Further improvement in cryo-ET data processing is still significant and urgent for more precise analysis and higher throughput. Here are some examples of the current problems. The limitation of the number of fiducial markers may lead to over-fitting in motion correction and tilt series alignment (Fernandez and Li, 2021). The reason for over-fitting is that the data from marked regions would be less than the imaging model’s parameters in proportion. Segmentation and particle picking also rely, to some extent, on manual labeling based on prior knowledge (Pyle and Zanetti, 2021). Image denoising can help particle picking and be the initial steps of an STA analysis. In later steps, since the coordinates are known, particles are extracted and used from noisy data. Furthermore, denoised data can be used to produce an initial model. Full-resolution data can be used for structural analysis to reach the highest resolution as possible (Taylor et al., 2006). While the experimental data in cryo-ET are progressively enriched, computational methods ought to leverage such abundant information for higher-resolution restoration and more accurate analysis.

**Figure 2:**
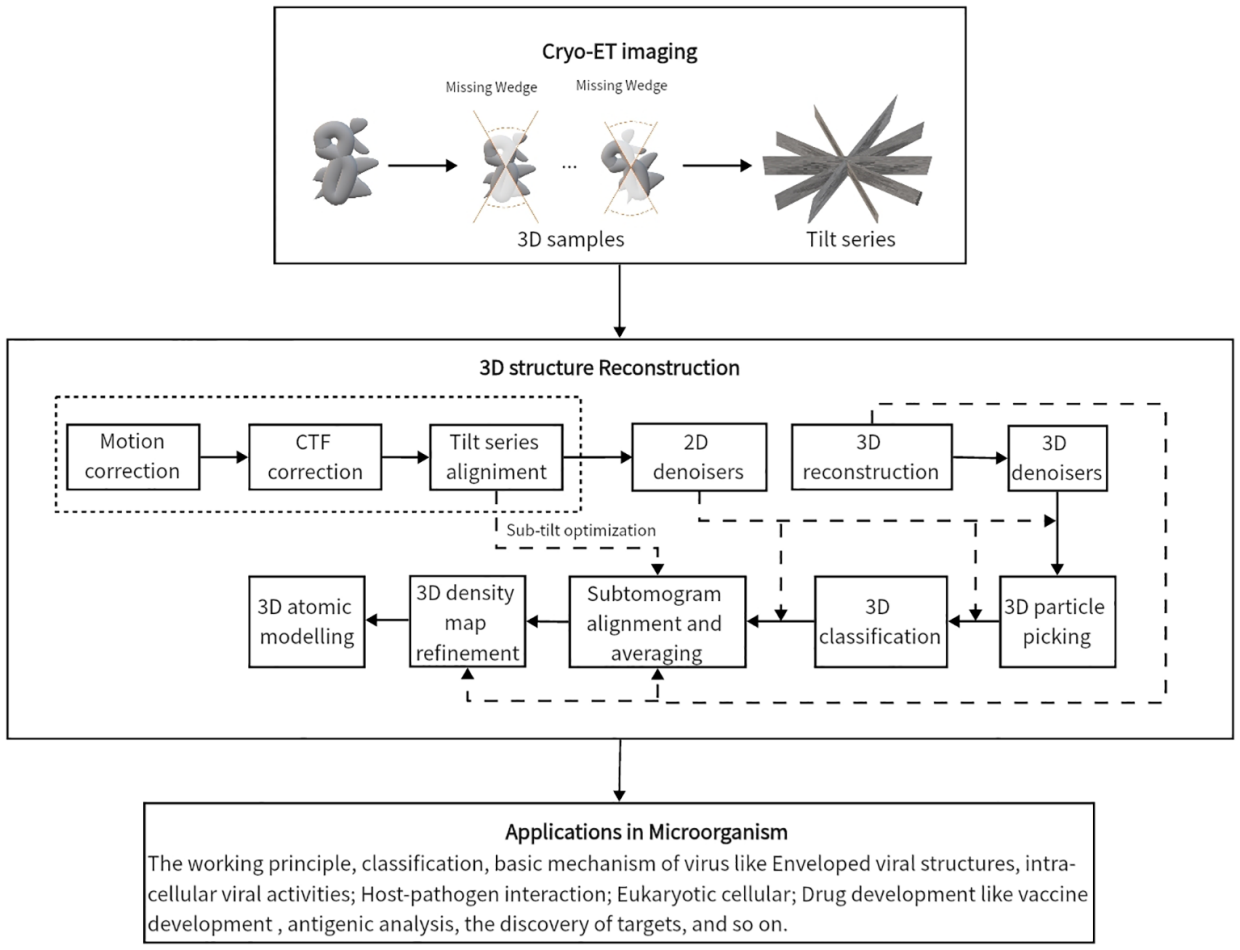
The general flowchart of the 3D reconstruction from tilt-series projections by cryo-ET. From top to bottom, the first frame shows the imaging process by cryo-ET. Then, the tilt series are the inputs of the second frame to reconstruct a 3D structure. The three parts in the dashed box are sometimes handled together. The last frame displays some applications to the microorganism.

This review summarizes current methods of artificial intelligence and mathematics for reconstructing 3D density maps and atomic models from two-dimensional (2D) tilt-series projections in cryo-ET. Here, we draw a general flowchart to show about 10 steps of 3D reconstruction in cryo-ET in **Figure 2**. It is worth noting that the process is subjected to appropriate adjustments and changes owing to differences in biological samples. The three steps in the dashed box in **Figure 2**, namely, motion correction, contrast transfer function (CTF) correction, and tilt-series alignment, are sometimes chosen to group into a program (Fernandez and Li, 2021). A denoiser is used to remove noise. While removing noise, it also removes the high-resolution signal. Thus, a denoiser is just used for some initial steps. The 2D denoiser is just an option to remove noise in 2D tilt-series projections, and 3D denoisers can remove noise in 3D subtomograms. The 3D denoiser can help improve the accuracy of 3D particle picking and classification. For the same classification, the subtomogram alignment and averaging (STAA) restores the 3D volumes by averaging to remove noise. Post-processing, also called refinement, corrects the 3D density map. Finally, the last step is building an atomic model from a 3D density map. In addition, denoising can be used for particle picking, initial averaging, and alignments, and classification. For higher-resolution information, the full-resolution volumes should be used for STA, alignments, and 3D density map refinement. Moreover, for real high resolution in STA analysis, sub-tilt optimization will take place. The methods for each step are shown in detail in the following sections, arranged as follows.

### 1.1 Organization of this paper

The 3D reconstruction in cryo-ET roughly contains 10 steps. Sections 2 to Section 11 show motion correction, correction of CTF, tilt series alignment, 2D and 3D denoisers, 3D reconstruction, 3D particle picking, classification, subtomograms alignment and averaging, post-processing for 3D density map, and atomic structure building in turn, respectively. We discuss the difficulties of each small task, the methods, and the prospects for the future. Section 12 discusses cryo-ET reconstruction research’s current status, difficulties, and future research prospects.

## 2 Motion correction

Motion happens because the samples are exposed to the electron beam, which will cause unsatisfactory blurs and high-resolution informational losses. This motion is called beam-induced motion. The electronic beam is produced through the acceleration of electrons at high voltage (approximately 300 kV). In cryo-electron tomography, the beam-induced motion is usually attributed to multiple factors, including electron beam interaction with the specimen, stage vibration, sample deformation, intense ionization in materials, and measurement error (Naydenova et al., 2020; Thorne, 2020). The local sample motion induced by the electron beam can be observed as a “doming” motion (Ganguly et al., 2022), where the sample suffers an upward deformation perpendicular to the sample plane. The intermolecular correlation shifts and the measurement error of the projections should be considered. Therefore, these motions must be corrected to reduce their impacts on low SNR and artifacts.

In cryo-electron tomography, a frame is a single image recorded by the direct-electron detectors. A movie is a sequence of frames during the electron beam exposure of the biological specimens (Li et al., 2013). Simulating the beam-induced movement and correcting the movie/frame data are an inverse problems in a mathematical sense. As the complex electron–sample interactions presented above, some mathematical models are developed based on the anisotropies. The two-dimensional or polynomial spline interpolation and stochastic processes are capable of fitting. In TomoAlign (Fernandez et al., 2018; Fernandez et al., 2019), the algebraic equation includes the 3D deformation of the sample as the motion correction through the different tilts as homogeneous polynomial vectors in the 3D-axis of a microscope, ${\text{D}}^{i}\left(r_{j}\right)\;=\;({\text{Δ}}_{x}^{i}\left(r_{j}\right),\;{\text{Δ}}_{y}^{i}\left(r_{j}\right),\;{\text{Δ}}_{z}^{i}\left(r_{j}\right))$ , where *r_j_* = (*x_j_,y_j_,z_j_*) are the *j*th fiducial marker’s coordinates in the axis of the sample stage, and *i* is the index of the image of the tilt series. In RELION 4.0 (Zivanov et al., 2022), the stochastic process estimation of the electron beam-induced motions is derived within Gaussian processes, which is an analogous method in RELION 3.0. Specifically, the motion of the interest particle is modeled on the path through the tilt series in 3-dimensional space. The optimization of parameters comprises the maximum likelihood of the posterior estimation determining the motion pattern and the irradiate damage accumulation. With the assumption that the sparse fiducial movements locally estimate the whole motion of the sample, it follows the limitation of the dependence of fiducial markers. The number of markers may contribute to an over-fitted approximation of the parameters introduced by polynomials.

For the particles of growing interest in marker-free alignments, such as cryo-lamella, Aretomo (Zheng et al., 2022) provides an integrated marker-free solution in a fully acquired tilt series for correcting the beam-induced motion. Aretomo regards the drift paths of feature in patches as the beam-induced local deformation throughout the whole tilt series. The rotation of the sample stage in cryo-electron microscopy can essentially be described by 3D rigid-body motion. By matching of this motion with the features, residuals of the original and matched samples represent the local deformation. Informed about the measured inter-subframe shifts, MotionCor2 (Zheng et al., 2017) describes these non-coherent and idiosyncratic shifts in the projection plane as smooth two-dimensional polynomials, gives least-squared estimates of correlated shifts between adjacent subframes, and maps backward to the damage-weighted image. The patches are artificially divided acquired images and the features are respectively extracted from them. MotionCor2 v1.5.0 is used to match the centers of these patches in the tilt series and detect features for local motion correction. Warp (Tegunov and Cramer, 2019) takes a similar process strategy as MotionCor2 but with no assumption on the 2D polynomials, where two grids decompose the global and local motion sources and perform motion correction separately. The dose-fraction frames are aligned and corrected in Fourier space. Instead of manually or semi-manually labeling marker locations, SparseAlign (Ganguly et al., 2022) is a mathematical method for marker localization and deformation estimation simultaneously. The SparseAlign solves a few deformation parameters based on an image-based loss between the forward projection of markers and the observed projections. Software such as TomoAlign (Fernandez and Li, 2021), Aretomo (Zheng et al., 2022), MotionCor2 (Zheng et al., 2017), Ximpp (Strelak et al., 2021), Warp (Tegunov and Cramer, 2019), RELION (Zivanov et al., 2022), and Unblur (Grant and Grigorieff, 2015) all provide solutions for motion correction.

Motion correction is indeed a vital step in ensuring the successful analysis of cryo-ET data. Most methods of motion correction in cryo-ET are based on those in cryo-EM. Consequently, many of these methods may not be specifically optimized for cryo-ET. In contrast, the different sample preparation, electron dose, and relative issues are supposed to be in consideration of image processing. It is a valuable recognition that algorithms correct beam-induced motion in the electron microscope or specimen stage rather than in the projection plane. More accurate tilt series alignments, tomography reconstruction, and subtomogram analysis will be carried out with considerable motion correction. However, the calculating limitations should be taken into account for the experimental approaches of raw image acquisition, such as the number of fiducial markers or intrinsic features and more accurate but omitted factors of beam-induced deformation beyond doming motion. Sometimes, the performance of motion correction is also limited by the low SNR and the high-tilt angles.

## 3 Determination, correction, and refinement of CTF

During the imaging process in cryo-ET, the low electron dose, the thickness of the specimen, and the tilt angles result in intricately noisy and blurred projections. The aberration of the lens and the defocusing give rise to the CTF, which restricts the recording of the high-resolution information (Marabini et al., 2015). The CTF causes periodic signal inversion over a range of frequencies and completely loses information on signals at zero-crossings (Turonova et al., 2017). Furthermore, the tilted images in cryo-ET have defocus gradients perpendicular to the tilt axis, resulting in different defocus values for different angles of projections. Therefore, CTF determination and correction are very significant steps before further reconstruction processing.

**Figure 3:**
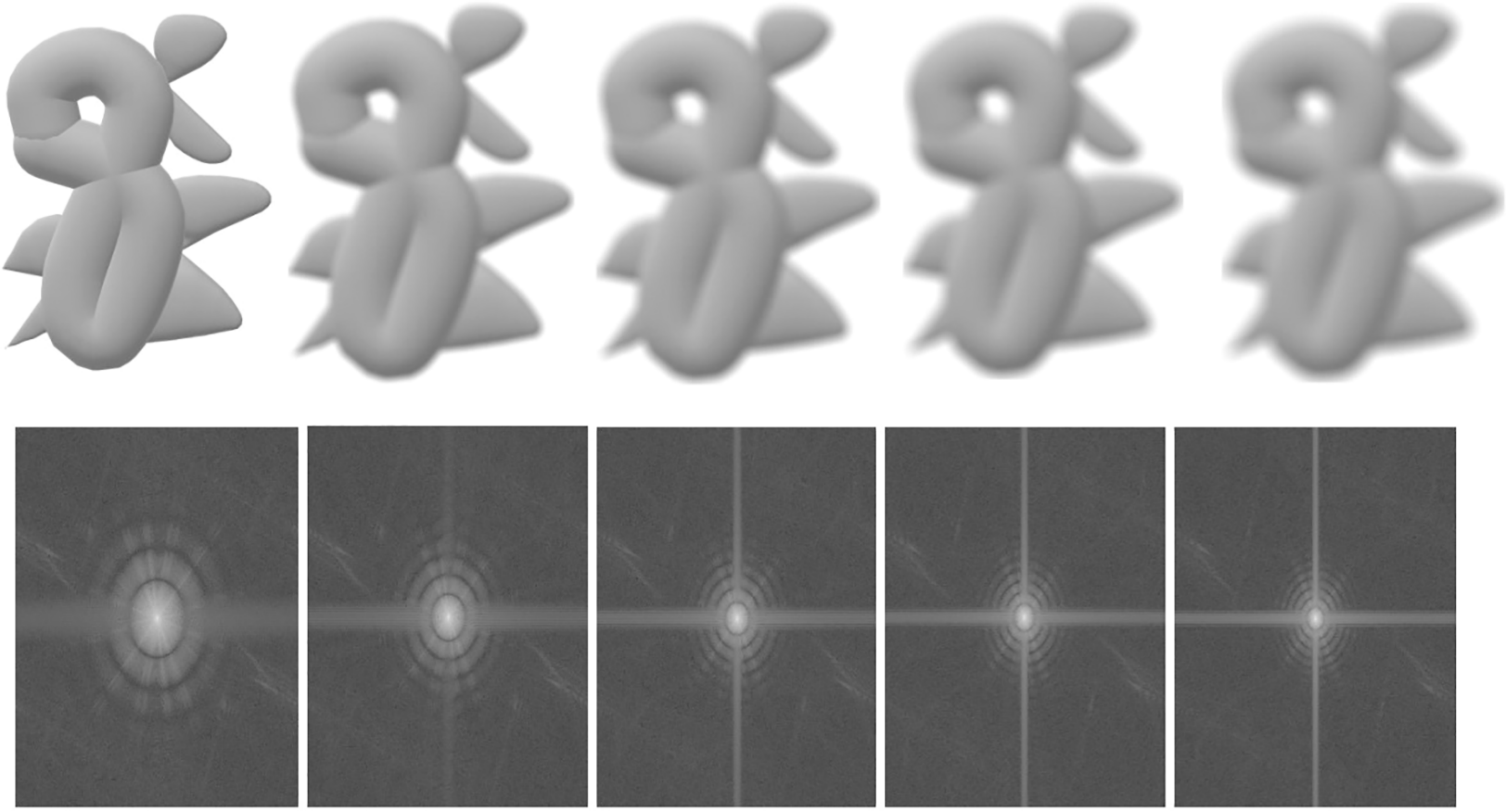
An illustration of the effects of the CTF and PSF on the 2D projected images. First row: blurred images; second row: the magnitude spectrum. From left to right, the first ones are original, and the others are increasingly blurry images and their corresponding magnitude spectrum.

The CTF is an oscillatory function, which is more severe at high frequencies and defoci. These oscillations cause changes in contrast and spectrum amplitudes for projections in Fourier space, which weakens high-resolution information. The CTF is affected by the defocus, spherical aberration, the wavelength of electrons, etc. (Voortman, 2014). From a physical point of view, the CTF (Zhang, 2016; Kunz and Frangakis, 2017) is defined as


(1) $$CTF\left(\text{t}\right)\;=\;\sqrt{1\;-A^{2}}\sin\left(\gamma \left(\text{t}\right)\right)\;-A\cos\left(\gamma \left(\text{t}\right)\right),$$

where *γ*(***t***) = *πλt* ^2^(Δ*z* − 0.5*λ* ^2^ *t* ^2^ *C_t_*), *t* is the modulus of the spatial frequency ***t***, *λ* is the electron wavelength, *C_t_* is the spherical aberration constant, Δ*z* is the defocus, and *A* is the amplitude contrast coefficient. The Fourier transform of the projected tomography is multiplied by the CTF to obtain the Fourier transform of the observed image, i.e.,


(2) F(f) =CTF(t) · F(I),

where *f, I* are the observed image and the underlying projection, and F is the Fourier transform. The effect of the CTF on the images’ magnitude spectrum is shown in **Figure 3**. The Fourier inverse transformation of the CTF is called the point spread function (PSF), denoted as *A*, i.e., F ^−1^(*CTF*(***t***)) = *A*(***x***) (Croxford et al., 2021). The PSF in real space blurs projection images as **Figure 3** shows.

Most existing literature mainly focuses on the methods of CTF determination and correction in Fourier space, which contains 2D-CTF and 3D-CTF determination and correction. Furthermore, these CTF correction methods usually combine with other steps like tilt series alignment and 3D refinement (Fernandez and Li, 2021). The 2D CTF correction approaches include e2ctf in EMAN2 (Tang et al., 2007), CTER (Penczek et al., 2014), CTFFIND4 (Rohou and Grigorieff, 2015), Gctf (Zhang, 2016), and goCTF (Su, 2019). The CTER method (Penczek et al., 2014) can estimate the parameters of CTF rapidly and accurately. These advantages imply that the CTER can spend less time analyzing high-throughput data and obtain more accurate results than other methods. The CTER can also provide an error assessment which is a cross-resolution correlation 1D function, which evaluates the similarity of the 3D structures with CTF correction or not. In 2015, the effects of 2D CTF parameters and correction methods were tested (Marabini et al., 2015) among ctfind, xmipp, appion, e2ctf, spider, and eman, but not for goCTF and Gctf. It shows that most CTF correction methods have minor differences even though each approach has its unique advantage. In Marabini et al. (2015), relatively speaking, the CTFFIND4 (Rohou and Grigorieff, 2015) has more stable and better results. CTFFIND4 (Rohou and Grigorieff, 2015) defined a specific formula for the spatial frequency to estimate CTF. Gctf (Zhang, 2016) is a GPU-accelerated global and local CTF estimation program based on astigmatism rotational averaging and self-consistency verification. In Gctf, Δ*z* = *z*(*θ*) = *z_u_* cos^2^(*θ* −*θ_ast_*)+*z_v_* sin^2^(*θ* −*θ_ast_*), where *z_u_, z_v_* are maximum and minimum defocus, and *θ_ast_* is the angle between *z_u_* axis and *x*-axis. The goal in Gctf is


(3) $$\left({\hat{z}}_{u},\hat{z_{v}},{\hat{\theta }}_{ast}\right)=\arg\underset{\left(z_{u},z_{v},\theta_{ast}\right)}{\max}\left\{CC\left(ln(\left|F\left(\text{t}\right)\right|\;-\;Bg\left(ln\;|F\left(\text{t}\right)|\right),\;|CTF_{sim}(\text{t})|\cdot e^{-\frac{B}{4}|\text{t}{|}^{2}}\right)\right\},$$

where *F*(***t***) is the amplitude spectrum, *Bg* is the calculated background, *CTF_sim_* (***t***) is the simulated CTF, *B* is the *B*-factor, and CC is the cross-correlation. Another method, GoCTF (Su, 2019), is a geometrically optimized approach for estimating the global focus gradient and local focus refinement per particle. The typical cryo-EM SPA approaches for the CTF correction do not take the defocus gradient into consideration.

There are some 2D correction CTF methods designed for cryo-electron tomography. The method in Fernández et al. (2006) is a classical method for CTF correction. Only a part of this method is implemented in the software CTFFIND3. To be specific, the defocus *D*(*x*) at any images of tilt series can be determined by the simple formula, i.e., *D*(*x*) = *D*_0_ + *d*(*x*)tan(*θ*), where *D*_0_ is the defocus at the untitled plane, d(x) is the distance to the tilt axis and *θ* is the tilt angle. Then, the CTF is estimated by the defocus, and its inverse Fourier transform is used to correct the particular tilted projection. An extended image acquisition method (Eibauer et al., 2012) is proposed to determine CTF by recording two high-dose images along the tilt axis for each tilt angle. Galaz-Montoya et al. (2016) proposed a per-particle CTF correction method for a single particle in cryo-ET, which automatically computes CTF for each particle and tilt-series images. This method proposes a linear regression strategy to fit the defocus gradient and further perform CTF correction. The Bsoft (Heymann, 2018) software is a tool including 2D-CTF correction for reconstructing macromolecular structures. The other software and methods for 2D CTF correction are shown in **Table 1**.

**Table 1:** A summary of methods and software for each reconstruction step in cryo-ET.

Reconstruction Steps	Software	Methods
Motion Correction	Relion (Zivanov et al., 2022) Ximpp (Strelak et al., 2021) TomoAlign (Fernandez and Li, 2021) Warp (Tegunov and Cramer, 2019)	Aretomo (Zheng et al., 2022) MotionCor2 (Zheng et al., 2017) Zorro (McLeod et al., 2017) Alignparts lmbfgs (Rubinstein and Brubaker, 2015) Unblur (Grant and Grigorieff, 2015)
Determination, Correction and Refinement of CTF	**2-dimensional CTF:** IMOD (Zivanov et al., 2019) emClarity (Fernandez et al., 2019) Relion (Jensen and Kornberg, 2000) EMAN2 (Tang et al., 2007) Bsoft (Heymann, 2018) Ximpp (Strelak et al., 2021) **3-dimensional CTF:** TomoAlign (Fernandez and Li, 2021)	**2-dimensional CTF:** goCTF (Su, 2019) Gctf (Zhang, 2016) CTF determination (Eibauer et al., 2012) CTFIND (Rohou and Grigorieff, 2015) e2ctf (Tang et al., 2007) Per-particle-based CTF-correction (Galaz-Montoya et al., 2016) **3-dimensional CTF:** 3D CTF (Kunz and Frangakis, 2017) NovaCTF (Turonova et al., 2017) ERDC (Croxford et al., 2021)]
Tilt Series Alignment	IMOD (Mastronarde and Held, 2017) PEET (Nicastro et al., 2006) Dynamo (Scaramuzza and Castaño-Díez, 2021) TomoAlign (Fernandez and Li, 2021) EMAN2 (Murray et al., 2014) AuTom-dualx (Han et al., 2019b)	Aretomo (Zheng et al., 2022) ClusterAlign (Seifer and Elbaum, 2022) Joint-mark-free-alignment (Han et al., 2019a) AuTom (Mastronarde and Held, 2017) RAPTOR (Ding et al., 2015) MarkerAuto (Han et al., 2015) UCSF tomo (Castaño-Díez et al., 2012) Protomo (Zheng et al., 2007)
3D Reconstruction	IMOD (Mastronarde and Held, 2017) EMAN2 (Murray et al., 2014) ICON (Deng et al., 2016) emClarity (Ni et al., 2022) AuTom-dualx (Han et al., 2019b)	IsoNet (Liu et al., 2022b) New iteration method (Zhai et al., 2021) Monte-Carlo-based method (Moebel and Kervrann, 2020) JDLMID (Ding et al., 2019) Image inpainting (Trampert et al., 2018) Regularization-based (Albarqouni et al., 2015) NUFFT (Chen and Förster, 2014) sMAP-EM (Paavolainen et al., 2014) FBP (Zeng, 2012) CRM (Han et al., 2021) LoTToR (Zhai et al., 2021) MBIR (Deng et al., 2016) DIRECTT (Kupsch et al., 2015) PSRT (Turoňová et al., 2015) WSIRT (Wolf et al., 2014) ASART (Wan et al., 2011) WBP (Carazo et al., 2006) MSIRT (Wan et al., 2009) SIRT (Trampert and Leveque, 1990)
Detection, Segmentation, 3D Particle picking	emClarity (Ni et al., 2022) Dynamo (Scaramuzza and Castaño-Díez, 2021) IMOD (Mastronarde and Held, 2017) Xmipp (Strelak et al., 2021) EMAN2 (Murray et al., 2014) Chimera (Henderson, 2013) SPHIRE-crYOLO (Sanchez-Garcia et al., 2018) pyTOM (Hrabe et al., 2012)	COS-Net (Zhou et al., 2021) Yolov3 (Wu et al., 2022) U-net-based (Zhou et al., 2023) TomoTwin (Rice et al., 2023) VP-Detector (Hao et al., 2022) (Hajarolasvadi et al., 2022) MemBrain (Lamm et al., 2022) Active learning method (Mo et al., 2021) Deepfinder (Moebel et al., 2021) A 2D-to-3D framework (Zeng et al., 2022)
Classification	PEET (Heumann et al., 2011) Dynamo (Scaramuzza and Castaño-Díez, 2021) RELION (Bharat and Scheres, 2016b) EMAN2 (Murray et al., 2014) emClarity (Ni et al., 2022)	SHERC 2021 (Gubins et al., 2021) SHERC 2020 (Zhao et al., 2018) Respond-CAM (Zhao et al., 2018) A multi-path CNN (Luo et al., 2019) DSRF3D-v2, CB3D (Che et al., 2018) MRA (Chen et al., 2014)
2D and 3D Denoisers	**2D Denoisers:** 2D-Topaz (Bepler et al., 2020) **3D Denoisers:** 3D-Topaz (Bepler et al., 2020)	**2D Denoisers:** ACE (Shi and Singer, 2022) *β*−GAN (Gu et al., 2022) NT2C (Li et al., 2022a) TV-based variants (Pang et al., 2020) Noise2void (Krull et al., 2019) Noise2Noise (Bepler et al., 2020) CWF (Bhamre et al., 2016) BM3D (Maggioni et al., 2013) **3D Denoisers:** SC-Net (Yang et al., 2021) 3D Noise2void (Krull et al., 2019) BM4D (Maggioni et al., 2013) A Monte Carlo Framework (Moebel and Kervrann, 2018)
Subtomograms Alignment and Averaging	PEET (Nicastro et al., 2006) emclarity (Ni et al., 2022) Dynamo (Scaramuzza and Castaño-Díez, 2021) pyTOM (Hrabe et al., 2012) EMAN2 (Murray et al., 2014) Relion (Bharat and Scheres, 2016a)	AItom (Zeng and Xu, 2020) AV3 (Bharat et al., 2015) Jim-Net (Zeng et al., 2021) Gum-Net (Zeng and Xu, 2020) STOPGAP (Wan et al., 2020) TomoMiner and TomoMinerCloud (Frazier et al., 2017) Reference-free alignment (Chen et al., 2013) High-through STA (Xu et al., 2012) MCCF (Leigh et al., 2019) MLE (Stölken et al., 2011)
Post-Processing for 3D density map	Relion (Zivanov et al., 2019) emClarity (Ni et al., 2022) EMAN2 (Murray et al., 2014) M (Tegunov et al., 2021) Servalcat (Yamashita et al., 2021) Phenix (Terwilliger et al., 2019; Terwilliger et al., 2020)	IsoNet (Liu et al., 2022b) DeepEMhancer (Sanchez-Garcia et al., 2021) LocSpiral (Kaur et al., 2021) LocalDeblur (Ramírez-Aportela et al., 2019) LocalScale (Jakobi et al., 2017)
Atomic Structure Building	HADDOCK2.4 (Neijenhuis et al., 2022) Phenix (Afonine et al., 2018)	Auto-DRRAFTER (Ma et al., 2022) CR-I-TASSER (Zhang et al., 2022) DEMO-EM (Zhou et al., 2020) *A* ^2^-net (Xu et al., 2019) CERES (Liebschner et al., 2021) CDMD(Igaev et al., 2019)

3D CTF correction methods for cryo-ET also exist, like NovaCTF (Turonova et al., 2017), TomoAlign (Fernandez and Li, 2021), 3D CTF (Kunz and Frangakis, 2017), and Entropy-regularized deconvolution (ERDC) (Croxford et al., 2021). The NovaCTF (Turonova et al., 2017) is a popular procedure for CTF correction in cryo-ET, which realizes CTF correction in each image with different defocus many times and improves reconstruction resolution. TomoAlign (Fernandez and Li, 2021) is also famous. The TomoAlign procedure realizes CTF correction and tilt-series alignment simultaneously, where the results of 2D CTF correction and tilt-series alignment in IMOD can be the initial inputs. To be specific, the defocus of the *i*th image in tilt series is *D^i^* (***p***) = *p_x_* sin(*θ^i^*) + *p_z_* cos(*θ^i^*) + *D^i^* (***o***), where ***p*** = (*p_x_, p_y_, p_z_*) is the particle coordinates with the center *o* of the tomogram, *D^i^* (***p***) and *D^i^* (***o***) are the averaged defocus and the defocus of the *i*th image, and *θ^i^* is the tilt angle. The estimated CTF is determined by the defocus. Three-dimensional CTF (Kunz and Frangakis, 2017) is performed on each slice of the reconstruction instead of the micrographs, which is based on the Jensen-Kornberg scheme. The ERDC approach by Croxford et al. (2021) is a deconvolution process based on entropy regularization in real space, where the regularization plays a role of robustness for noise.

Both 2D and 3D CTF correction approaches, the accurate estimation of parameters in CTF is crucial and challenging. With the increase in cryo-ET data and the development of artificial intelligence algorithms, it may be possible to estimate the parameters of physical formulas for CTF by network methods in the future. At present, there are still many gaps before the network approach is applied to CTF. For instance, there is no benchmark dataset and no ground truth to verify the accuracy of CTF correction methods.

## 4 Tilt series alignment

The probability of misalignment of the projection is induced by the distortions during tilt series acquisition, including shifts, rotations, translations, and magnifications. The misalignment of the projections would hamper the SNR enhancement and 3D restoration in the following workflow. Tilt series alignment is an essential component of procedures modifying the impacts of several kinds of distortions by modeling the projections onto 2D images. In addition, full tracking in every tilt angle allows more complete data collection at high magnification, which can be applied to various tilt strategies, including the dose symmetric scheme. Specifically, the dose-symmetric tilt series scheme (Hagen et al., 2017) starts at zero degrees’ tilt and moves up to the highest tilt in both tilt directions simultaneously. This method concentrates high-resolution information in the lower tilts where the sample is thinner, thus providing maximum information transfer. With the assistance of full tracking, the dose-symmetric tilt series scheme avoids resolution limitations, improves information transfer, and minimizes the effect of sample distortions.

**Figure 4:**
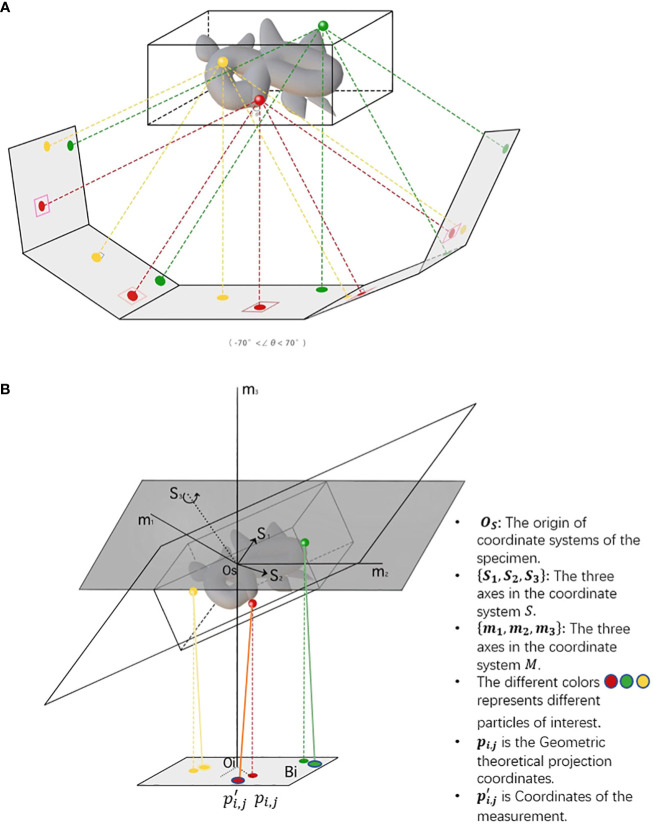
The imaging of cryo-ET tilt series. **(A)** A sample diagram of obtaining tilt series by projecting specimens in cryo-ET, where the tilt angles range from −70^◦^ to 70^◦^. **(B)** A three-coordinate system of obtaining tilt series in cryo-ET. The relative deviation between theoretical and measured coordinates *p_i,j_* and ${p^{\prime }}_{i,j}$ . The different colors, namely, red, green, and yellow, represent different particles of interest. In the projection plane, there are theoretical and measured coordinates of features/fiducials.

After motion correction, CTF correction and coarse tilt series alignment, a more precise projection model is needed for fiducial-based alignment and feature-based alignment. For fiducial alignment and calibration, “gold beads” in sample preparation are tiny gold nanoparticles used as fiducial markers. Geometrically, once the gold beads are placed as common fiducial markers in sample preparation for precise alignment, it follows the coordinate systems of the specimen, microscope, and each projection image, *S* = {*O_s_*,**s**_1_,**s**_2_,**s**_3_}, *M* = {*O_m_*, **m**_1_, **m**_2_, **m**_3_}, *B_i_* = {*O_i_*, **u** *_i_*, **v** *_i_*}. In **Figure 4**, the geometric projection formula of a position in the sample onto the *i*th image (Hanssen, 2018) can be represented as:


(4) $$\begin{array}{c} p_{i,j}=R\left(\gamma_{i}\right)PR\left(\beta_{i}\right)R\left(\alpha_{i}\right)D_{i}\left(m_{i},s_{i},\delta_{i}\right)r_{j}+d_{i},\\ i=\;1,\ldots ,N\;\;\;\;,j=\;1,\ldots ,N_{m}, \end{array}$$

where *N* is the number of images of tilt series, *N_m_* is the number of fiducial markers, **p** *_i,j_* = (**u** *_i,j_*, **v** *_i,j_*) is the coordinate of the projection of the *j*th fiducial marker in the *i*th view, r*_j_* is the coordinate in *M* of the *j*th fiducial marker, **d** *_i_* = (Δ*u_i,j_*,Δ*v_i,j_*)*^T^* is the shift relative to *O_m_*, *P* denotes the matrix for projection onto (**u**, **v**) plane, *D_i_* denotes the matrix representing specimen changes with respect to the scaling factor *m_i_*, the additive scaling factor *s_i_*, the skew rotation *δ_i_* and multiplicative factor *t_i_*, and *R*(*γ_i_*)*,R*(*β_i_*)*,R*(*α_i_*) are rotation matrices around the *z*-, *y*-, and the x-axis of *M*. By minimizing the total error relative to measured projection ${\text{p}}_{i,j}^{'}$ and estimated projection p*_i,j_* (Hanssen, 2018),


(5) $$\underset{a_{i},\beta_{i},\gamma_{i},m_{i},s_{i},\delta_{i},{\text{r}}_{j},{\text{d}}_{i}}{\arg\min}\sum_{i=j}^{N}\sum_{j=1}^{N_{m}}w_{i,j}\cdot \rho ({\text{p}}_{i,j}^{'}-{\text{p}}_{i,j}).$$

The over-fitting problem is common in fiducial-based alignments because the number of parameters is relatively large for the numbers of fiducial markers as well as the limited number of tilt series, albeit there are techniques for reducing parameters and making the model more robust. In TomoAlign (Fernandez and Li, 2021), the projection matrix is reduced and the motion-aware polynomials are induced as a term on the right side of Eq. (4), **p** *_i,j_* = **M** *_i_* **r** *_j_* + **S** *_i,j_* + **d** *_i_*, where **M** *_i_* = *m_i_ **P*** **R** *_i_*, **R** *_i_* including the rotation matrix around three axes in *M*, ${\text{S}}_{i,j}=\;\left({\text{S}}_{i,j}^{u},{\text{S}}_{i,j}^{v}\right)$ denoting the beam-induced motion undergone by the sample in the *i*th image, which corresponds to a number of parameters by giving a different type of the doming deformation polynomials or splines. Specifically, the doming motion causes an upward rise at the center of the carbon hole in the electron microscope grid while the periphery remains relatively unchanged.

Feature-based alignment is a classical marker-free alignment approach suitable for lamellae, which is recognized for practicality due to the fewer requirements for sample preparation and the broader scenarios, such as the prohibition of the fiducials. The difficulties in feature localization, extraction, and matching exceed similar procedures in fiducial-based alignments like ClusterAlign (Seifer and Elbaum, 2022). In contrast, the substantial number of virtual markers contributes a statistically robust and accurate alignment with relatively expensive computation. Satisfactorily, several automatic platforms and programs show their performance on feature-based alignments and joint alignments like AuTom (Mastronarde and Held, 2017), AuTom-dualx (Han et al., 2019b), and a joint method (Han et al., 2019a). The AuTom package contains marker-free (Han et al., 2014)and marker-based (Han et al., 2015) alignment. The marker-free method (Han et al., 2014) is based on Scale-Invariant Feature Transfrom (SIFT), which realizes more accurate feature extraction, more stable feature description, and more robust feature matching and tracking. The marker-based method is a fully automatic alignment of data with fiducial markers (Han et al., 2015). AuTom-dualx (Han et al., 2019b) is a tool for marker-based alignment of cryo-ET dual-axis tilt series. At first, the two tilt series are dealt independently with detecting and tracking of fiducial marks, and bundle adjustment for single-axis data. Then, the two tilt-series results are combined by transforming their axes to obtain global projection parameters and tracks by solving a non-linear least square problem. Finally, the last stage is global parameter optimization of tilt series based on a simultaneous reconstruction. The standard method in Han et al. (2019a) is an iterative approach for marker-free alignment, which combines track-based and intensity-based alignment. Some software also provide automated tilt-served alignment like IMOD (Mastronarde and Held, 2017), EMAN2 (Murray et al., 2014), and Dynamo (Scaramuzza and Castaño-Díez, 2021).

The traditional approaches in marker-free alignments are dedicated to calculating the shift for the highest cross-correlation in the Fourier space, determining the common line of each 2D projection based on the Fourier slice theorem and the projection matching by constructing a 3D model as an intermediate reference. Sometimes, the traditional approaches serve as coarse alignment and preprocessing schemes. Aretomo (Zheng et al., 2022) utilizes the traditional marker-free methods with stages of tilt-angle offset determining planarity and recursion, in-plane rotational alignments, translational alignments, and local alignments with patches, which reduces the impacts of the distortions above progressively at sufficient accuracy.

## 5 2D and 3D denoisers

**Figure 5:**
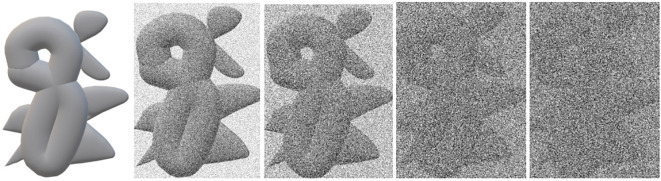
An illustration of the effect of noise on an image. From left to right, the left one is an underlying image; the others are noisy images as the noise becomes larger and larger.

The electron dose is strictly limited due to radiation damage to the biological samples (Han et al., 2022), which is an essential factor for noise. Electron beam intensity and exposure time are factors to control the final exposure dose on the sample. During the imaging process, tilt angles usually vary from −70^◦^ to 70^◦^, which causes a missing wedge (Moebel and Kervrann, 2018). In other words, the parts of projections will be missing since the specimen cannot be rotated to plus/minus 90^◦^. As the tilt angle increases, so does the ice thickness that the electrons have to penetrate. Low electron dose and thick ice are two significant factors to lower the SNR. **Figure 5** shows the effect of noise on an image.

Noise in cryo-ET is a critical factor in limiting the resolution of three-dimensional reconstructed structures (Frangakis, 2021). In the reconstruction processing, Aligning and averaging of the same particles is a well-known approach to remove noise and enhance the signal. However, the STAA step is appropriate for a number of homogeneous macromolecules, not for intrinsic molecular flexibility and dynamics with a continuum or discrete changes of conformation, such as proteins in folding, antibodies, and lipoproteins (Han et al., 2022). The intrinsic molecular flexibility and dynamics needs some denoisers for resolution enhancement. Denoisers usually remove noise and enhance the image contrast in the low frequencies. High noise, complicated noise types, and no ground truths are three factors that make the denoising task in cryo-ET complex (Yang et al., 2021). These three factors make most existing denoisers unsuitable for tomographic images. Next, we introduce 2D and 3D image denoisers in detail.

2D denoisers are applied to tomographic projections, where we focus on relevant mathematical methods and deep learning methods. Natural images are taken by the camera according to the sensitive unit, like images taken by mobile phones. Here, 2D projection images by cryo-ET are unnatural images. The existing mathematical methods for denoising natural images include total variation (TV)-based variants (Pang et al., 2020), block matching and 3D collaborative filtering (BM3D) (Maggioni et al., 2013), k-singular value decomposition (k-SVD) (Aharon et al., 2005), and traditional Wiener filter (TWF). However, the types and levels of noise in the tomography of cryo-ET are different from those of natural images, which cannot obtain a satisfying result on tomographic projections (Gu et al., 2022). The imaging principle of cryo-EM and cryo-ET is the same. However, compared with cryo-EM, cryo-ET needs to project specimens under different view angles and assign electronic dose for limiting radiation damage. Thus, 2D projection images in the tilt series by cryo-ET have a lower SNR than 2D projections by cryo-EM. To date, there are few 2D denoisers specifically designed for cryo-ET, while many 2D denoisers are designed for cryo-EM. Some researchers applied the methods of denoising images for cryo-EM to 2D projection images in cryo-ET as a preprocessing step. Mathematical models have been developed for denoising images in cryo-EM in the past few years. The noise of 2D projections is usually assumed to follow Gaussian distribution, while the actual noise is complicated. Although these assumptions for mathematical modeling are simplified relative to the actual situation, the experimental results are acceptable. A mathematical approach named Covariance Wiener Filtering (CWF) (Bhamre et al., 2016) was proposed for denoising images and correcting the CTF of 2D projection images simultaneously. In the method, the imaging model in Fourier space is *Y_i_* = *A_i_X_i_* + *ξ_i_* for *i* = 1,2*,…,n*, where *Y_i_,A_i_,X_i_,ξ_i_* are the 2D observed image, spread point function, 2D clean image, and noise. The CWF approach estimates the mean *µ* and covariance Σ of the underlying clean images, chooses Fourier Bessel basis to represent the images, and then obtains restored images by the Wiener filter based on $\hat{\mu }$ and $\hat{\Sigma }$ instead of estimating the spectral SNR in TWF. The CWF method takes projection directions, ice thickness, and structural variability into account in Σ. The estimation $\hat{\mu }$ can be calculated by


(6) $$\hat{µ}\;=\text{ arg }\underset{\mu }{\text{min}}\left\{\sum_{i=1}^{n}∥Y_{i}-A_{i}\mu {∥}_{2}^{2}+\lambda ∥\mu {∥}_{2}^{2}\right\},$$

where *λ >* 0 is the regularization parameter. The estimation $\hat{\Sigma }$ can be calculated by


, (7) $$\hat{\Sigma }\;=\text{ arg }\underset{\Sigma }{\text{min}}\left\{\sum_{i=1}^{n}∥A_{i}\Sigma A_{i}^{T}+\sigma^{2}I-(Y_{i}-A_{i}\mu )+{\left(Y_{i}-A_{i}\mu \right)}^{T}{∥}_{F}^{2}\right\}$$

where *σ* ^2^ is the variance of Gaussian noise. After obtaining $\hat{\mu }$ and $\hat{\Sigma }$ , the restored images can be solved by the Wiener filter as follows:


(8) $${\hat{X}}_{i}=\;\left(I-H_{i}A_{i}\right)\hat{µ}+H_{i}Y_{i},$$

where $H_{i}=\;\hat{\Sigma }A_{i}^{T}{\left(A_{i}\hat{\Sigma }A_{i}^{T}+\sigma^{2}I\right)}^{-1}$ is the Wiener filter (Frappart et al., 2016). In 2022, an improved version of the CWF method named Ab-initio Contrast Estimation (ACE) is proposed (Shi and Singer, 2022), which improves contrast estimation. The approach is derived under the imaging model *Y_i_* = *c_i_A_i_X_i_* + *ξ_i_*, where *c_i_* is the unknown contrast of the clean image. In the ACE method, *c_i_* makes the estimation of $\hat{\Sigma }$ more accurate and the resolution of the repaired images higher. Although the CWF and ACE methods can realize an improved result, this issue still has much room for improvement due to their strict assumptions.

Next, we turn to deep learning methods for 2D denoising. Many deep learning-based methods have a satisfying performance on denoising in cryo-ET (Frangakis, 2021). The Generative Adversarial Network (GAN) is a network for generating images. GAN consists of a generator and a discriminator, which are neural networks. The generator tries to generate fake data and cheat the discriminator. The discriminator distinguishes the real and fake data. The generator and discriminator are neural networks that run in competition during training. In 2018, the *β*−GAN approach with *l*_1_ and *l*_2_ loss (Gu et al., 2022) was proposed for denoising 2D cryo-EM images. To overcome the lack of ground truths, Noise-Transfer2Clean (NT2C) (Li et al., 2022a) applies the simulation software InSilicoTEM (Vulović et al., 2013) to produce simulated 2D projection images in cryo-EM. There are three parts in NT2C: noise extraction, noise modeling, and denoiser training. There is the coarse convolutional neural network (CNN) denoiser and the refined CNN denoiser, which are connected by fusing the noise in real noisy images to the simulated clean images through the contrast-guided noise and signal re-weighting step. NT2C can realize many denoising results, but its operation is tedious, and its results seriously depend on simulated clean images. Furthermore, some deep learning-based methods do not rely on ground truths, such as Noise2Noise (N2N) (Lehtinen et al., 2018) and Noise2void (N2V) (Krull et al., 2019). The Topaz method (Bepler et al., 2020) is a version of N2N, which is for 2D denoising. This method contains four network structures, including affine, fully connected neural network (FCNN), small U-net convolutional network, and U-net convolutional network. The loss function is a measure of evaluating the difference between the predicted results by the algorithm and the real dataset. The loss function is usually reduced during training the network. Its loss can be *l*_0_, *l*_1_, and *l*_2_ as follows:


(9) $$\arg\underset{\text{Θ}}{\min}\left\{E_{x_{a},x_{b}∼X}∥f\left(x_{a}\right)-x_{b}{∥}_{i}\right\},$$

where *i* = 0,1,2, and *x_a_, x_b_* are noisy neighboring odd and even frames, *f* is the network, *X* represents the distribution of images, and E represents mathematical expectation. At the overall results, the U-net can realize a better result. However, the model’s training data cannot cover all cases of noisy images in cryo-EM. From another point of view, the losses of the Topaz model are simple, and maybe one can replace the losses with an adaptive loss based on real data.

3D denoisers can be applied to tomograms. The popular methods include Block-Matching and 4D filtering (BM4D) (Maggioni et al., 2013), a Monte Carlo Framework (Moebel and Kervrann, 2018), 3D Noise2Noise (3D-Topaz) (Bepler et al., 2020), 3D Noise2void (Krull et al., 2019), and the Sparsity Constrained Network (SC-Net) (Yang et al., 2021). BM4D was designed for denoising a single 3D natural image. One can apply BM4D directly to a single noisy volume image of cryo-ET. The Monte Carlo Framework is a general denoising framework to overcome noise in cryo-ET. One can integrate any 3D Gaussian denoiser like BM4D into the framework. The Topaz method proposes two network frameworks, including U-net-3d-10a and U-net-3d-20a, where the difference is the average pixel size of training data 10 Å and 20 Å, respectively. The training data of the 3D Topaz method are based on the even/odd frames of 32 aligned cryo-ET tilt series and the training time is over 1 month. The 3D Topaz can usually realize a good denoising performance and is regarded as a benchmark 3D volume image denoiser in cryo-ET. The 3D Noise2void method is trained in the CNN on noisy images, which does not need noisy image pairs or ground truths. SC-Net is a self-supervised learning network with a sparsity constraint. The SC-Net is based on the 2D filter to produce the 2D reconstructed images and then apply the 3D reconstruction method to obtain smoothed 3D volumes to guide the network training. The inputs of SC-Net are a linear combination of four parts, including raw noisy volumes and smoothed volumes. The loss function of SC-Net contains volumetric reconstruction, sparsity-guided smoothing, expectancy constraint, and regularization. There are some comparisons among SC-Net, BM4D, N2N, and N2V on simulated and real-world data (Yang et al., 2021). SC-Net can realize competitive performances with Topaz.

Although the existing methods can achieve acceptable denoising results for some cases in cryo-ET, there are still room to improve resolutions. For 2D denoisers, most denoising methods are specially designed for cryo-EM, not cryo-ET. Because of the different image acquisition strategy between cryo-EM and cryo-ET, the noise in projections is different. On one hand, due to the constraints of the total electron dose, noise is higher in the per-tilt projection image recorded for cryo-ET. In comparison to cryo-EM acquisitions, the full dose is used to record only one image in zero tilt. On the other hand, as the tilt angle increases, so does the thickness of the ice and sample the electrons need to penetrate, prohibiting the recording of the high-resolution signal to these projection images. Hence, the 2D projections by cryo-ET is very noisy. We should be careful to apply 2D denoisers in cryo-EM, which are directly applied to denoise 2D projection images of tilt series in cryo-ET. The factors such as lower electron dose and changes in ice thickness should be considered. Designing more accurate 2D tomography denoisers according to different imaging conditions in cryo-ET is an urgent task in the future. For 3D denoisers, there are several popular methods for denoising 3D volume images in cryo-ET, including BM4D, Topaz, N2V, and SC-Net. BM4D is for a single 3D volume image. Topaz, N2V, and SC-Net are networks based on training data. The difficulties of denoising 3D volume images are no ground truths, high noise levels, and complex noise types. To address this, some imaging simulation algorithms for cryo-ET can be designed to fill up the shortage of no ground truths, or some novel 3D denoising network structures that do not need ground truths can be constructed. Alternatively, some adaptive 3D denoising mathematical models can be proposed in the future. Restoring 3D volume images in cryo-ET is a tough but important task for improving resolution.

## 6 3D reconstruction and reduction of missing wedge

**Figure 6:**
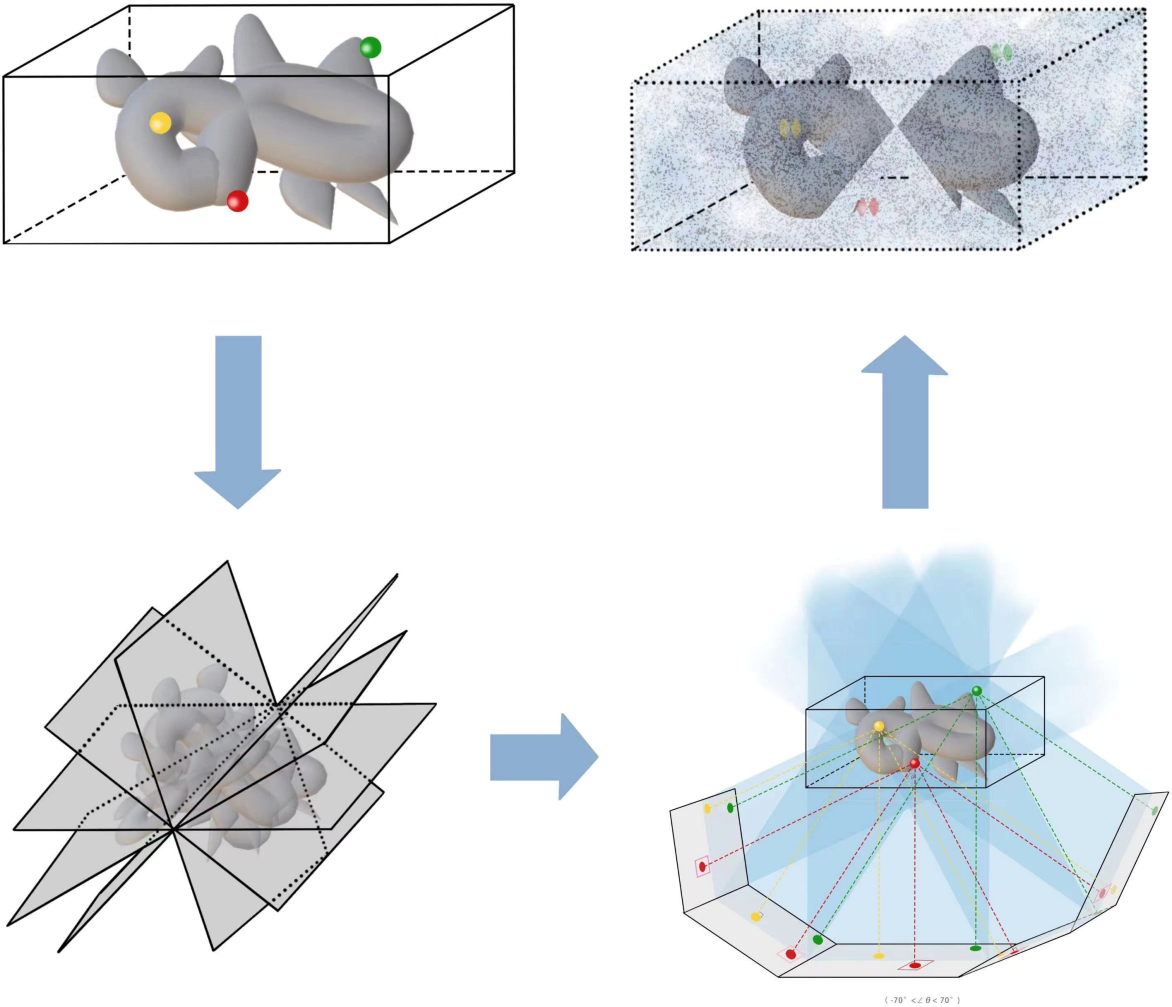
This is an illustration of the 3D reconstruction step from tilt series in cryo-ET, where the final volume suffers from the missing wedge.

The 3D reconstruction in cryo-ET aims to restore 3D volume images from 2D aligned images (Frank, 2005). Owing to the limitation of electron dose and constraint of tilt angles, low SNR and artifacts caused by the missing wedge are two difficult points to handle in cryo-ET (Ding et al., 2019); thus, many existing 3D reconstruction methods are not suitable for cryo-ET. **Figure 6** shows a simple illustration of 3D reconstruction step from the tilt series in cryo-ET, where the complete volume suffers from the missing data. In electron tomography (ET), the most common strategy to acquire data is the single-axis tomography (Han et al., 2017), although dual-axis tomography (Tong et al., 2006) is also used. Single-axis imaging rotates around the same axis during the imaging process, while dual-axis imaging rotates around two axes that are perpendicular to each other, which can obtain two different tilt-series images. The latter is designed to reduce the missing information to a “missing pyramid” experimentally. For the single-axis imaging process, the missing wedge (MW) information makes the reconstruction problem ill-posed. In some sense, MW can be restored by 3D reconstruction methods (Zhai et al., 2021). Then, the restored 3D volumes can be transported to 3D denoisers or 3D subtomogram image alignment and averaging to obtain high-resolution results. For the dual-axis imaging process (Han et al., 2019b), combining information from two tilt-series images is a difficult task.

Over the past few decades, many approaches have risen to deal with 3D reconstruction for the single-axis imaging process in cryo-ET. We firstly summarize the existing literature on 3D reconstructions proposed for the single-axis imaging process. The mathematical models can be divided into two categories: real space and Fourier space. The Fourier projection slice theorem (also known as the central section theorem) (Frank, 2005) is the key point of Fourier-space-based methods. The Fourier projection slice theorem tells us that the Fourier transform of 2D projection images equals the slice operator acting on the Fourier transform of 3D volume, whose mathematical expression is


(10) $$F_{2}\circ P\left(V\right)\;=S\circ \;F_{3}\left(V\right),$$

where *V* is a 3D volume, F*_i_* is the *i*−D Fourier transformation for *i* = 2,3, *P* is the 2D projection operator that integrates along the *z*-axis, and *S* is the slice operator that sets the component of the *z*-axis equal to 0. The Fourier transformation does not lose any image information. The Fourier-based approaches contain filtered back-projection (FBP) (Zeng, 2012), non-uniform fast Fourier transforms (NUFFT) (Chen and Förster, 2014), and the iterative compressed-sensing optimized non-uniform fast Fourier transform reconstruction (ICON) (Deng et al., 2016). The FBP method uses the same filter function for all projections. The ICON is a popular 3D reconstruction software based on NUFFT with compressed sensing and sparsity assumption in cryo-ET. In ICON, the imaging process is expressed as *f* = *Ax* + *N* in Fourier space, where *f* and *x* are the discrete Fourier transform of projections and the density of specimen, respectively, *A* is a non-uniform Fourier sampling matrix, and *N* is noise. Then, the mathematical model of ICON (Deng et al., 2016) is


(11) $$\arg\underset{x}{\text{ min}}\left\{∥Px{∥}_{L_{0}}\text{| }A^{H}WAx-A^{H}Wf{∥}_{L_{2}}<\epsilon \right\},$$

where *P* is a sparse transform matrix, *W* is the weight of nonuniform sampling, *A^H^* is the conjugate transpose of *A*, and *ϵ* is a constant.

In addition, there are many mathematical models in real space for 3D reconstruction in cryo-ET. Because of the missing wedge, the mathematical problems for 3D reconstruction are ill-posed. The most common methods include weighted back-projection (WBP) (Carazo et al., 2006), a regularization-based method (Albarqouni et al., 2015), the Monte Carlo approach (Moebel and Kervrann, 2020), sMAP-EM (Paavolainen et al., 2014), an iteration-based method (Zhai et al., 2021), and an image-inpainting-based method (Trampert et al., 2018). The WBP method applies different filters for different projections according to their geometric characters. For the iteration-based approaches, the imaging formation process can be simplified as *f* = *Ax* + *N*. Specifically, there are the algebraic reconstruction technique (ART) (Gordon et al., 1970), an adaptive simultaneous algebraic reconstruction technique (ASART) (Wan et al., 2011), the simultaneous iterative reconstruction technique (SIRT) by Trampert and Leveque (1990), the progressive stochastic reconstruction technique (PSRT) by Turoňová et al. (2015), direct iterative reconstruction of computed tomography trajectories (DIRECTT) algorithm (Kupsch et al., 2015), model-based iterative reconstruction (MBIR) (Deng et al., 2016), low-tilt tomographic reconstruction (LoTToR) (Zhai et al., 2021), and the constrained reconstruction model (CRM) (Han et al., 2021). The ART method aims to solve a large and sparse linear equation system, where one can obtain an approximate solution by Kaczmarz’s iterative method. There are some variants of the SIRT method that contain modified simultaneous iterative reconstruction (MSIRT) (Wan et al., 2009) and weighted simultaneous iterative reconstruction (WSIRT) (Wolf et al., 2014). The WSIRT method is a combination of the WBP and the SIRT methods. The CRM introduces a constrained term based on SART and SIRT. The minimization formulation of CRM (Han et al., 2021) is


(12) $$\underset{b_{i,}g_{i}}{\min}∥D_{i}b_{i}+B_{i}g_{i}-\;f_{i}{∥}_{2}^{2}\;\mathrm{s. t.}\;b_{1}=b_{2}=\ldots =b_{N},$$

where *b_i_* and *g_i_* are sample images and their relevant backgrounds, *D_i_* and *B_i_* are the image formation operators of *b_i_* and *g_i_*, respectively, and *f_i_* is their measured projection. The sparsity Kaczmarz’s algorithm solves the CRM problem. Another alternative approach is to regard missing wedge as an image inpainting problem (Trampert et al., 2018), which repairs the missing part with some general information about the images.

There are several neural network methods (Ding et al., 2019; Liu et al., 2022b) for 3D reconstruction and the reduction of missing wedge in the single-axis imaging process of cryo-ET. IsoNet (Liu et al., 2022b) is a neural network method for reconstructing the missing wedge information and increasing SNR. The IsoNet contains five steps: deconvolve CTF, generate mask, extract, refine, and predict steps. The refine step plays a crucial role in IsoNet. The refine step needs a pair of tomograms to train. However, the extracted subtomograms have missing wedge. The IsoNet adopts an iterative way to update the pairs with the predicted subtomograms, which takes advantage of deep learning to fill up the missing wedge by predicting information. The joint deep learning model (JDLMID) (Ding et al., 2019) is for the inpainting of the missing wedge and de-artifact. JDLMID tries to fill up the missing wedge by image inpainting. The JDLMID method contains two parts: GAN frameworks with residual in a residual dense block for inpainting and a U-net for eliminating artifacts. In the future, more neural networks for 3D natural image reconstruction can be revised to be suitable for 3D reconstruction in cryo-ET.

Besides the single-axis imaging process, the dual-axis imaging process (Mastronarde, 1997; Tong et al., 2006; Han et al., 2019b) is an acquisition strategy to collect more data and reduce the missing information to a “missing pyramid” in the experiment (Guesdon et al., 2013). However, the projections of the dual-axis tomograms have a lower SNR than the single-axis type, and matching two orthogonally oriented tilt series is a new and difficult task. For the dual-axis imaging process in cryo-ET, the Etomo (Mastronarde and Held, 2017) in the software IMOD and PEET can deal with the dual-axis data. Moreover, Han et al. proposed a new toolkit called AuTom-dualx (Han et al., 2019b) for the 3D reconstruction of dual-axis tomograms. The combination of both two tilt series in AuTom-dualx is based on fiducial markers. They designed a new algorithm to match the two tilt-series coordinates and then embedded it into the SART and SIRT methods for 3D reconstruction. The dual-axis tomogram (Winter and Chlanda, 2021) has analyzed the interactions of the Ebola virus and action-VP40.

In brief, there are two imaging processes: singe-axis and dual-axis. As shown above, many mathematical models and neural networks have been developed for 3D reconstruction in cryo-ET for the singe-axis imaging process. They aim to reconstruct 3D volumes from 2D projections and try to fill up the missing wedge by prediction. Some restored methods depend on prior knowledge and are not data-driven, like MTV (Albarqouni et al., 2015). Others rely on the known information of observed projections to restore the missing wedge (Liu et al., 2022b). Although the existing methods have realized some acceptable 3D density maps of specimens in some cases, there are some areas for improvement. The methods based on prior constraints may generate elongation, streaking, and ghost tail artifacts due to the lack of using the information of the acquired projections. The existing methods depending on the acquired projections may not obtain ideal reconstructed results from incomplete projection orientations. In the future, we hope that more well-performed restored-processing methods can be developed for reconstructing 3D density maps in cryo-ET image processing.

## 7 3D particle picking

**Figure 7:**
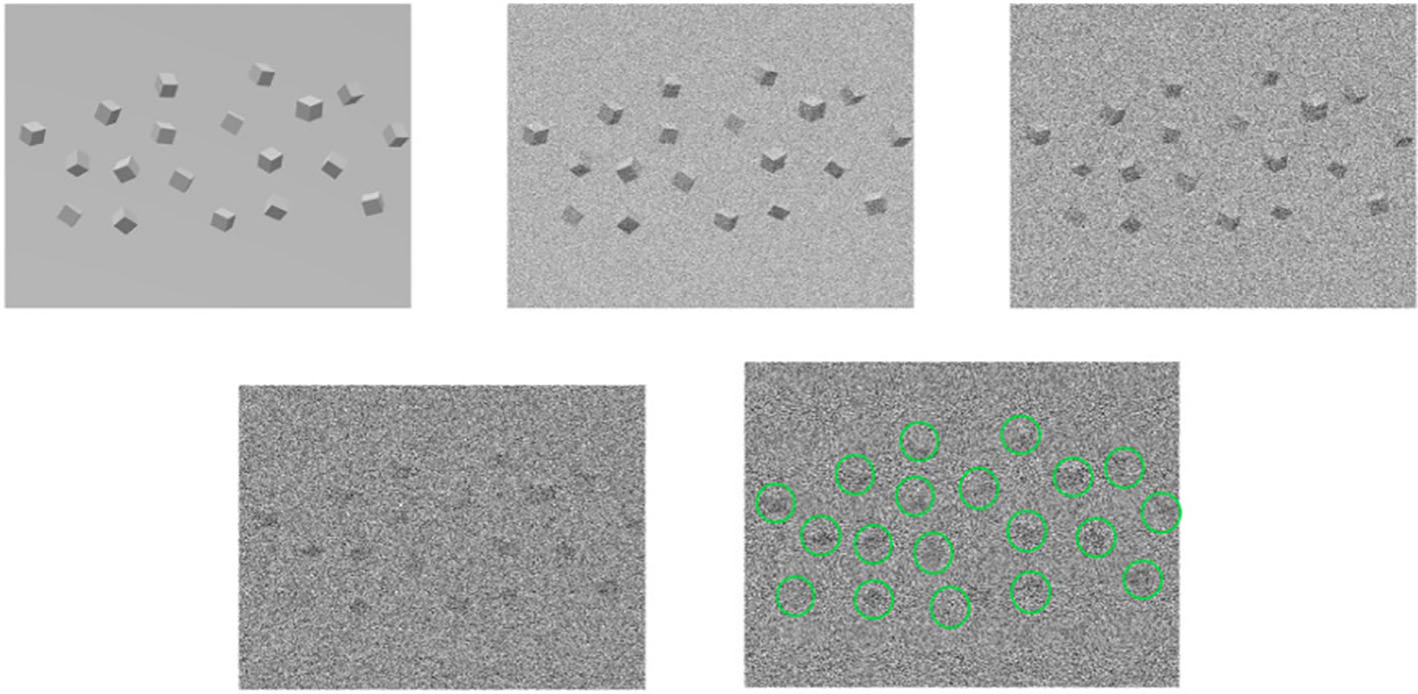
An illustration of the 3D particle packing task. For the first row, from left to right, the first one are the underlying particles. The second and the third rows are noisy tomograms as the noise becomes larger, so does the left one in the second row. The last one detects and marks particles in 3D particle picking.

**Figure 8:**
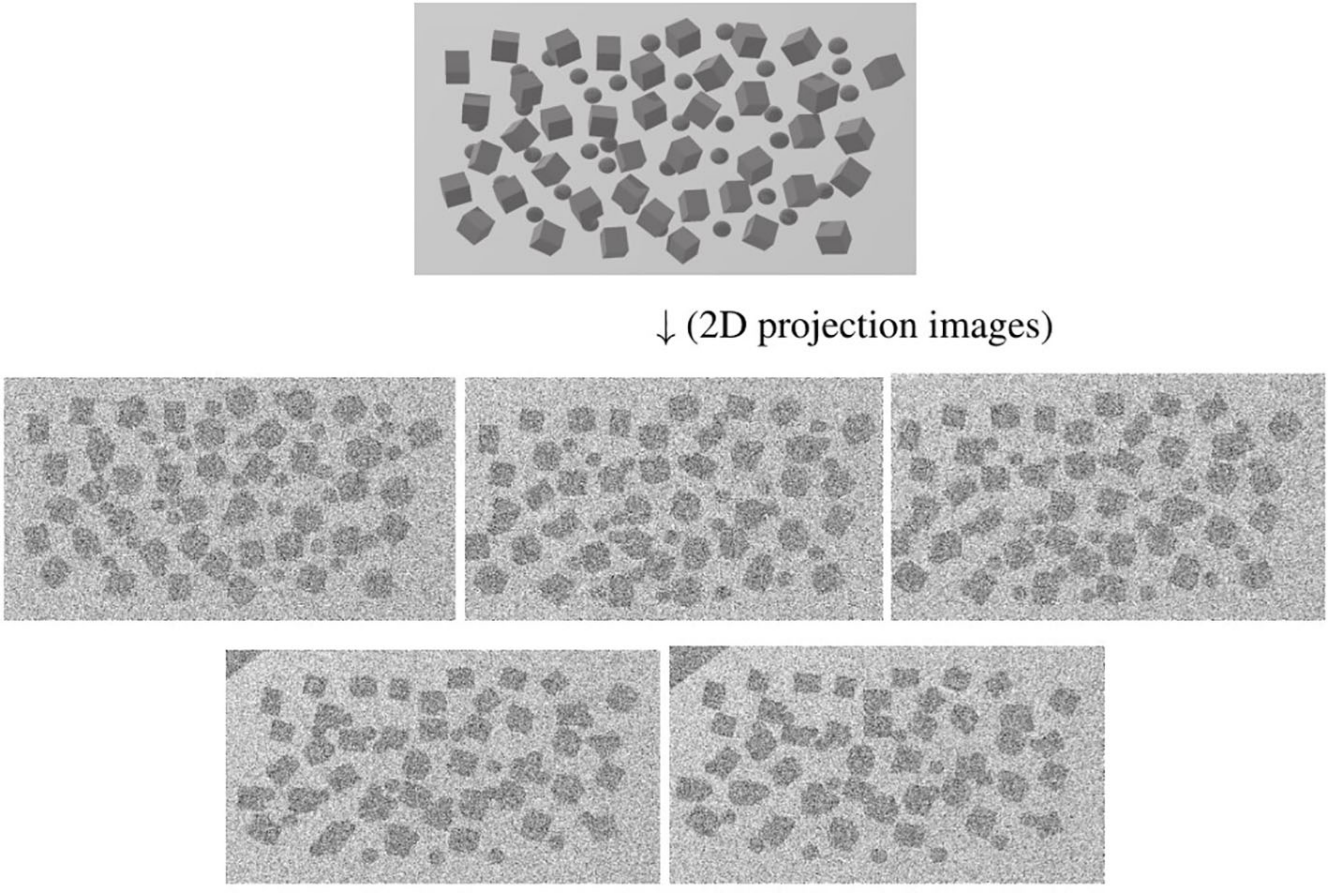
An illustration of simulation of 2D projection images in different tilt angles by rotating the biological sample. The top picture shows the particles located in an overcrowded environment. The other pictures are 2D projection images as the tilt angle increases from left to right and from top to bottom. The cubes stand for particles.

**Figure 9:**
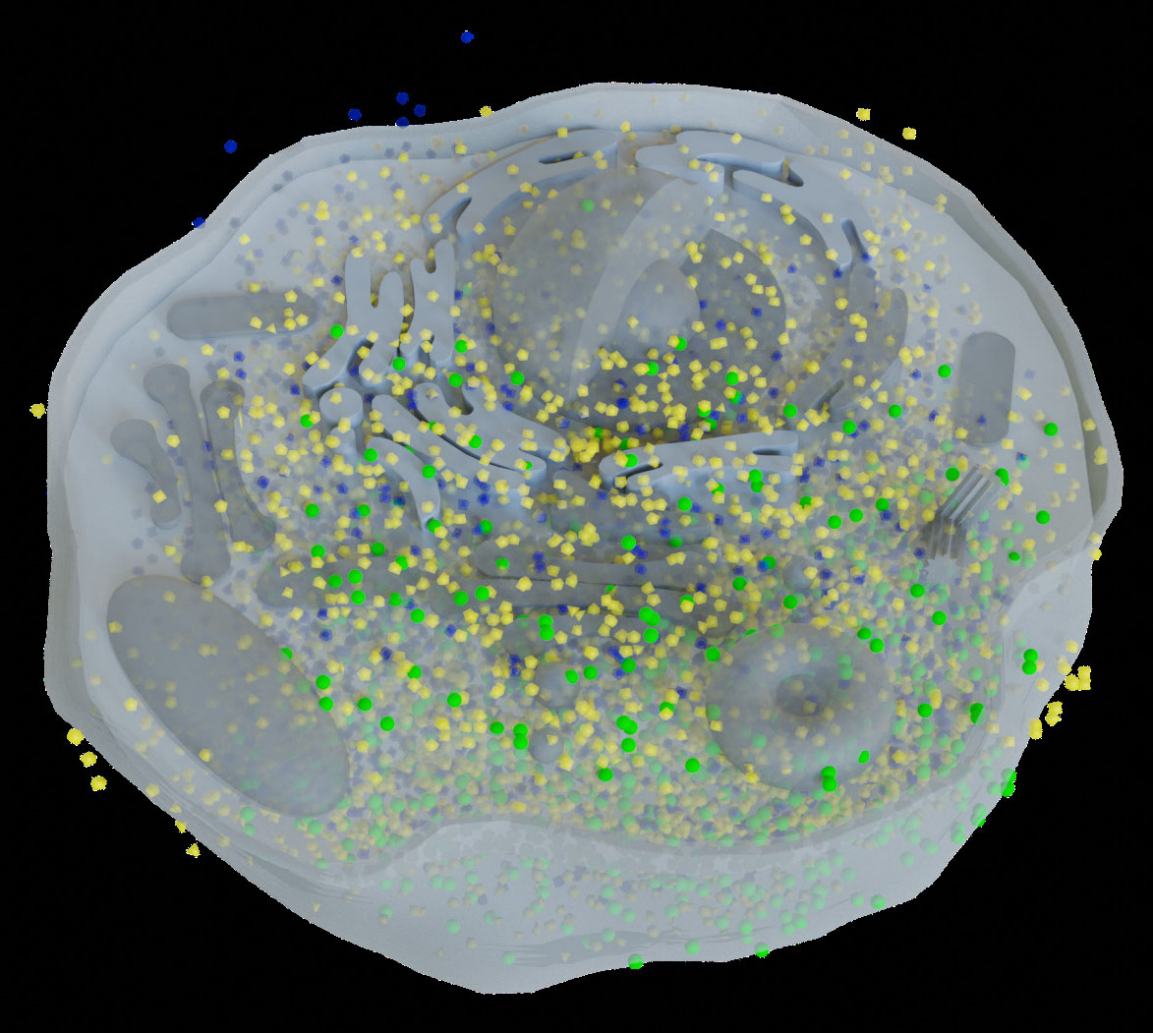
This is the biological environment *in situ* for picking macromolecular complexes (represented by green balls) inside a cell.

Since a 3D reconstructed tomogram contains multiple copies of specimens, the 3D particle picking task focuses on detecting and picking the particles of interest to biologists. As **Figure 7** shows, there are many noisy particles of different orientations. The higher the noise, the harder to detect the particles of interest. There are four factors that make 3D particle detection difficult: the low SNR in 3D reconstructed tomograms, missing wedge, the small sizes of particles, and the crowded environment (Castaño-Díez et al., 2017; Mo et al., 2021). **Figure 8** shows simulations of 2D projection images by rotating the biological sample, where the particles are located in an overcrowded environment. The overcrowded environment and the difficulties of picking particles *in situ* are shown in **Figure 9**, that is, for the case of particles inside the cells. The 3D particle picking task contains two tasks: detection and segmentation. The low SNR makes it impossible to detect particles by eyes and requires computational methods. The existing methods of particle picking usually focus on coordinate identification and extraction of 3D targeted particles in volumes. There are segmentation-based methods like instance segmentation (Liu et al., 2022a), CASSPER (George et al., 2021), and detection-based methods like one-stage detection (Liu et al., 2022a). These methods for picking 3D particles in cryo-ET are done in a manual or semi-automated pattern. Manual marking particles are empirical and time-consuming. In recent years, many methods based on artificial intelligence have been developed.

Recently, many new neural network methods have been developed, like those based on 3D-CNN, manifold, U-net, active learning, and so forth. It can be divided into template-based and template-free methods. For the former, the Dynamo provides a basic geometrical library, and users can select a specific one to do 3D particle picking. Here, we only focus on the latter. We collect some methods for detecting and segmenting particles. The MemBrain (Lamm et al., 2022) and 3D-UCaps (Hajarolasvadi et al., 2022) are designed for detecting membrane–protein complexes. The MemBrain is a deep learning frame combined with using CNN for scoring and means shift clustering. The 3D-UCaps combines 3D Capsule Network (CapsNet) and CNN to decrease the computing cost and improve accuracy. A learning method by integrating dense CNN and autoencoder into U-net (Zhou et al., 2023) is for the cell membrane segmentation. In addition, Pyle et al. (2022) propose several strategies for picking and cleaning membrane–protein complexes. The Deepfinder (Moebel et al., 2021) and TomoTwin (Rice et al., 2023) are designed for detecting macromolecules. The Deepfinder can identify multiple macromolecular complexes simultaneously by combining U-net, a 3D CNN-based model, and a clustering algorithm. TomoTwin maps particles into a high-dimensional space that is called the embedding space. It measures particles by embedding their representations. To save computing time, the YOLOv3 (Wu et al., 2022) regards particle picking as an object detection task using one-stage detection. To combine 2D detection and segmentation methods into 3D cases, a novel 2D-to-3D framework (Zeng et al., 2022) for detection is used by detecting objects firstly in 2D projections and then localizing objects in 3D structures. Active learning (Mo et al., 2021) is an active-learning-based approach designed with region proposal and Resnet3d under a new class label. There are some learning-based approaches, like the cryo-ET one-shot network (COS-Net) (Zhou et al., 2021). In addition, there is a new method to simulate crowded cells in cryo-ET (Pei et al., 2016) to check the effects of 3D particle picking methods in a crowded particle environment. Some software contains 3D particle picking like Dynamo (Castaño-Díez et al., 2017), EMAN2 (Murray et al., 2014), Bsoft (Heymann, 2018), TomoTwin (Rice et al., 2023), Deepfinder (Moebel et al., 2021), and VP-Detector (Hao et al., 2022).

In fact, 3D particle picking is a time-consuming and computing-consuming task. Because the sizes and shapes of particles vary, the existing methods have their cases for the application. How to select an appropriate method is a question that is worth exploring. In the future, embedding biological samples in manifolds can be further developed. However, finding a suitable embedding space and representation needs to be explored and experimented. Some novel neural networks can also be extended to 3D particle detection or segmentation in cryo-ET.

## 8 Classification

Biomolecules constantly change their structures and conformations to function in the cellular environment. Cryo-ET enables the discovery of these diverse conformations *in situ* (Zhang, 2019). Yet, classifying these conformations accurately is challenging due to low SNR, uncertain classification numbers, and imbalanced class proportions (Taylor et al., 2006). Additionally, the missing wedge introduces variations in the structural information, depending on the orientations of the biomolecules (Heumann et al., 2011).

Classification of the subtomogram is used to extract consistent structural features from heterogeneous conformations (Wan and Briggs, 2016). Specifically, the fundamental purpose of classification is to find multiple conformations and compositions of known complexes, and discover unknown complexes. Similar to tilt-series alignment and subtomogram alignment, classification also relies on cross-correlation optimization in the tomograms (Frangakis et al., 2002; Heumann et al., 2011; Hrabe et al., 2012). In addition, pattern recognition schemes, based on *a priori* knowledge, are commonly used in research to classify the particles of interest. These schemes based on *a priori* knowledge require high-resolution experimental structures to generate clusters that are subjected to imaging experimental parameters. At the same time, low SNR and missing wedge also limit the feature representation of clusters.

The current approaches to perform classification are simultaneous within subtomogram alignment or are based on *a priori* knowledge. On the one hand, when the classification is decoupled from the subtomogram alignment, the constrained principal component analysis (PCA) method based on K-means or hierarchical clustering is often used, which brings higher computational consumption as its voxel-by-voxel comparison. For the interested complex and its specific functional regions, the PCA method has limitations when dealing with data distributions that exhibit higher levels of heterogeneity. Generally, the binning method is used to reduce the dimensions of data. On the other hand, the comparison method with multiple references is often used in simultaneous alignment and classification. The multiple references approach (MRA) relies on *a priori* knowledge of existing high-resolution structures. Thus, it can achieve higher cross-correlation coefficients by minimizing the probability of maximum likelihood estimation. The noise outside regions of structural change can reduce classification accuracy. To address this limitation, MRA was developed for inter-reference structural differences (Chen et al., 2014). Although the MRA is theoretically computationally simpler, it requires iterative operations to achieve classification convergence, which requires more demanding multi-reference sampling. Software packages for classification are available in Dynamo (Scaramuzza and Castaño-Díez, 2021), RELION (Bharat and Scheres, 2016b), emClarity (Ni et al., 2022), and EMAN2 (Murray et al., 2014; Chen et al., 2019).

In recent years, bioinformatic deep learning methods have significantly improved the classification of heterogeneous subtomogram of interest particles. The tomoDRGN (Powell and Davis, 2023) can reconstruct a heterogeneous ensemble of structures by learning a new neural network including a variational autoencoder (VAE) and decoder. This network can learn a continuous low-dimensional representation of structural heterogeneity in cryo-ET data (Powell and Davis, 2023). A learning-based approach for sub-break classification is proposed in Xu et al. (2017), addressing the challenges of complex data distributions and scalability. This approach combines deep learning for supervised feature extraction with unsupervised clustering and template-free classification, offering a complementary solution to traditional methods. Thus, it improves the performance of classification based on deep learning of three designed CNN models for large-scale systematic macromolecular structures. The Respond-weighted Class Activation Mapping (Respond-CAM) algorithm presents a method for extracting and interpreting significant structural features by CNNs (Zhao et al., 2018). An adversarial domain adaptation method is developed to enhance the discovery of new structures and facilitate data recovery in the context of imbalanced classification data (Luo et al., 2019). A combined data processing strategy addresses the challenges of imbalanced classification data and the composition of real molecules, which demonstrates significant improvements in classification and segmentation performance (Che et al., 2018). SHREC is an online 3D Shape Retrieval Challenge that has seen the development of novel deep learning-based methods for classifying cryo-electron tomograms. SHERC 2020 (Zhao et al., 2018) and 2021 (Gubins et al., 2021) evaluated classification results from six and seven groups, respectively. In SHERC 2021, methods like URFinder, DeepFinder, U-CLSTM, MC DS DS Net, YOPO, CFN, and TM-T/TM-F were proposed, with MC DS Net showing superior performance overall.

Classification is an essential step in the whole procedure, which provides input for subtomograms alignment and averaging. Although there are satisfying results for classification, better methods are still needed for higher accuracy and precision. In addition, ContinuousFlex (Harastani et al., 2022) is specifically designed to analyze continuous conformational variability. However, the continuous conformational variability of macromolecules still poses a challenge in this field.

## 9 Subtomogram alignment and averaging

After the classification of 3D particles (subtomograms), the next step is usually aligning and averaging 3D particles of the same class to denoise (Castaño-Díez and Zanetti, 2019; Zhang, 2019). It is worth noting that STAA is suitable for static conformational variability of macromolecules not for heterogeneous structures. The heterogeneous structures mean that there exists discrete or continuous conformational variability of macromolecules. The heterogeneous macromolecules can be denoised by the denoising algorithms in Section 5. Random orientation and low SNR are two factors that make the STAA task difficult in cryo-ET. There are two keys in subtomograms aligning which are 3D translations and rotations (Leigh et al., 2019). Because of the large shapes of 3D tomograms, computing cost and time should be considered. In this section, we summarize the existing approaches to the STAA problem.

The existing methods for STAA can be divided into three classes: maximum likelihood estimation (MLE) (Stölken et al., 2011) in real space, maximum cross-correlation function (MCCF) (Leigh et al., 2019), and neural networks (Zeng and Xu, 2020). Because the MLE method needs to go through all pixels of 3D subtomograms, the computational cost and time of the MLE method are relatively high. For speeding up the calculation of the MCCF-based methods in real space, there are some fast aligning approaches based on common gradient (Xu and Alber, 2012) and stochastic average gradient (SAG) (Lü et al., 2019) by parallel optimization. The multi-reference alignment (Zhao et al., 2022) is a simple mathematical model for particles alignment and averaging. Furthermore, some fast rotational correction-based aligning methods have been developed by the translation-variant rotational matching in Fourier space under some constraints (Xu et al., 2012). A dissimilarity function of 3D volumes (Förster et al., 2008) has been defined to obtain the 3D translations and rotations iteratively. A constrained cross-correction (CCC) was proposed to reduce the missing information to a “missing pyramid”. The spherical cross-correlation function of Chen et al. (2013) can be regarded as an extension of the MCCF method. There is a Fourier space-constrained fast volumetric matching based on Fourier space equivalence of constrained correlation measure (Xu et al., 2012). There is an improved STA method based on biophysical analysis and supramolecular context (Metskas et al., 2022). Some software can realize the task of STAA, such as Relion (Bharat and Scheres, 2016a), Dynamo (Scaramuzza and Castaño-Díez, 2021), Emclarity (Ni et al., 2022), STOPGAP (Wan et al., 2020), AItom (Zeng and Xu, 2020), and TomoMiner and TomoMinerCloud (Frazier et al., 2017).

In recent years, some methods based on neural networks have arisen for the task of STAA, like Gum-Net (Zeng and Xu, 2020) and Jim-Net (Zeng et al., 2021). The Gum-Net is a geometric unsupervised matching network only for 3D subtomogram alignment. The Gum-Net is based on 3D discrete cosine transform (DCT) for feature extraction, spatial transformer network for alignment, and Pearson’s correlation as the loss to train (Zeng and Xu, 2020). The Jim-Net is an end-to-end unsupervised CNN for the two tasks of cluster and alignment simultaneously (Zeng et al., 2021). There are two parts of the Jim-Net, namely, an aligning process based on constrained cross-correlation loss and a clustering process based on the Gaussian mixture model (GMM). The neural network methods can be better realized when the training sets are large.

In the STAA task, 3D subtomogram aligning is challenging and highly time-consuming. The existing methods have applied parallel optimization in real space and variant translation in Fourier space to speed up the computing process. The neural networks can realize a good result for the STAA that is based on the train data. Some methods for 2D particle alignment and averaging in cryo-EM can be generalized to the 3D STAA in cryo-ET, such as invariant-rotation features: first-, second-, and third-order autocorrelation (Singer, 2019; Marshall et al., 2020). Some manifold learning methods by Kileel et al. (2021) for dimensionality reduction may be considered to solve the STAA task.

## 10 Post-processing for 3D density map

Owing to the imaging noise, radiation damage, the motion of the samples, and biases in the above reconstruction steps, it is necessary to correct the details in the reconstructed 3D density maps (Rosenthal and Henderson, 2003). The post-processing procedure is used to modify the loss of contrast and fill in the details in the restored 3D density maps to visualize high-resolution features as the final reconstructed step. IsoNet (Liu et al., 2022b) is a deep learning method containing the refinement part for 3D reconstructed density maps, which has already been introduced in Section 5. The post-processing step is used to restore details of 3D density maps, in both cryo-EM and cryo-ET. Here, we summarize post-processing methods into three kinds, namely, map sharpening (Rosenthal and Henderson, 2003), density modification (Terwilliger et al., 2019), and refinement (Urzhumtsev et al., 2022).

Firstly, map sharpening can be roughly divided into global and local approaches. The global sharpening methods for the full volumes are almost based on *B*-factor correction (Rosenthal and Henderson, 2003). There is also an adjusted surface area measure (Terwilliger et al., 2018) for global sharpening by combining details and connectivity of the maps. The resolutions of the whole reconstructed volumes are usually not the same, which means some parts of 3D volumes are restored well while others are flawed. The local sharpening approaches for raw subtomograms are designed for local restoration, including LocalDeblur (Ramírez-Aportela et al., 2019), LocalScale (Jakobi et al., 2017), LocSpiral (Kaur et al., 2021), an adjustment of density maps in Phenix (Terwilliger et al., 2018), and DeepEMhancer (Sanchez-Garcia et al., 2021). The LocalDeblur is a Wiener filter for image deblurring based on local resolutions of the original maps. The LocSpiral provides a method to compute the local *B*-factor by the spiral phase transform. The LocalScale and Phenix need atomic maps as references, which can help to obtain good results. However, the requirement of atomic maps is a limitation to use these two approaches. The training data of the DeepEMhancer are pairs of experimental maps and post-processed maps by the LocScale. The well-trained DeepEMhancer is applied to reconstructed maps and realizes better results than the global sharpening methods. Secondly, the density modification (Terwilliger, 2000; Terwilliger, 2001) utilizes the maximum likelihood estimation based on prior knowledge and other known macromolecular structures. The improved procedure by Terwilliger et al. (2019); Terwilliger et al. (2020) needs two independent half-maps and some prior information of the reconstructed maps as inputs which available in the Phenix software. This method is suitable for those maps with preliminary knowledge or similar known macromolecular structures. Thirdly, there are some refinement methods for post-processing, like Servalcat (Yamashita et al., 2021), Relion (Zivanov et al., 2022), and M (Tegunov et al., 2021). The Servalcat computes a weighted *F_o_*− *F_c_* difference map, which is a part of REFMAC5 in the Collaborative Computational Project for electron cryo-microscopy (CCP-EM), where *F_o_, F_c_* is the Fourier transform of the observed map and calculated map from atoms. The weighted *F_o_*−*F_c_* difference map can help to enhance weak features like hydrogens. The refinement in Relion (Zivanov et al., 2022) contains CTF refinement and tilt-series refinement, which correspond to optical aberration and geometrical refinement separately. The M (Tegunov et al., 2021) software realizes refinement by a CNN denoiser for 2D images before 3D construction to improve local resolution. Furthermore, there is a framework by Burt et al. (2021) for three-dimensional reconstruction to multi-particle refinement in M, where it provides a link between the Dynamo and the Warp-Relion-M.

In short, the existing post-processing methods further optimize 3D volumes by calculating the whole or local *B*-factor, deep learning-based density modification, de-blurring, and denoising. In future, some representation methods and measures can be designed to describe the difference between density maps and atomic maps. In addition, as the 3D atomic structures increase, more new neural network methods can be applied to post-processing.

## 11 Atomic structure building

Biologists can analyze biological functions and conduct medical research through three-dimensional atomic structures (Xu et al., 2019). Atomic structure reconstruction (ASR) from density maps is significant. With the increase of atomic structure data and the improvement of computing equipment, artificial intelligence methods (Giri et al., 2022) have been applied to build atomic models automatically instead of by hand. Some deep learning methods have been summarized in Giri et al. (2022) for building atomic structures from density maps. To avoid repetition, we briefly introduce novel methods from two aspects of building and refining atomic models.

Some deep learning methods build atomic models using the training data of 3D density maps and the corresponding atomic structures. Amino Acid Network (*A* ^2^-net) (Xu et al., 2019) built an Amino Acid dataset of density maps. The *A* ^2^-Net contains two stages for detecting 3D amino acids and searching the leading chains of amino acids. It can determine a molecular structure rapidly and accurately. Recently, as the predicting structure methods with sequences develop, there appears some novel ASR methods that apply structure prediction as one of their parts. The inputs are usually 3D density maps and sequences. The Auto-DRRAFTER method by Ma et al. (2022) is designed to build an RNA model automatically based on the structure prediction method Rosetta. Based on the protein structure prediction I-TASSER method, the CR-I-TASSER (Zhang et al., 2022) identifies the templates from the Protein Data Bank (PDB) to promote atomic assembly. The CR-I-TASSER combines the I-TASSER folding simulations and the *C_α_* atom model by deep residual convolutional neural networks and molecular dynamics (MD) simulation to build the atomic structures. There are some similar methods, like domain-enhanced modeling using cryo-electron microscopy (DEMO-EM) (Zhou et al., 2020). The constructed atomic models can be refined further by fitting them to density maps such as an accurate real-space refinement (Urzhumtsev et al., 2022) and refinement in Phenix (Afonine et al., 2018). For the atomic structures, one can also perform interface refinement by HADDOCK2.4 (Neijenhuis et al., 2022) and refine further by correlation-driven molecular dynamics (CDMD) (Igaev et al., 2019). In turn, the CERES method (Liebschner et al., 2021) can refine 3D density maps by the constructed atomic models.

Above all, the existing methods can build or refine atomic models automatically. Although the existing methods can realize good results, there are some limitations like insufficient training data and the low resolution of density maps. Furthermore, as the structure prediction field develops rapidly, novel methods for the ASR can combine them into building atomic model procedures in the future.

## 12 Discussion

Structural biologists increasingly favor cryo-ET because of its ability to image large biological specimens *in situ*. The atomic structure of microorganisms is important to make drug discoveries, host–pathogen interactions, and so on. Publicly available databases like EMDB (Lawson et al., 2016) and EMPIAR (Iudin et al., 2016) separately provide 3D density maps and original 2D tilt-series projections. It involves a small amount of resolved density maps and corresponding tilt series applied in the microorganism in nature. However, the amount of data occupies a vast storage, which is a challenge for computer equipment. To the best of our knowledge, there is no unified benchmark of cryo-ET for deep learning method training, testing, and comparing. In future, computer hardware development for massive data storage and uniform standard training and test datasets will be needed.

With the development of computing technology and artificial intelligence, the resolution of resolved structures is increasing. Although many reconstruction software shown in **Table 1** have been developed, such as IMOD, RELION, DYNAMO, and EMAN2, each has its characteristics. However, there are several problems: (1) For structural biologists, after obtaining the cryo-ET tilt-series projections, selecting suitable software quickly and accurately remains a difficult problem; (2) Files of different software are in different formats, which cannot be used among different software. The ScipionTomo (Morena et al., 2022) provides an integrated platform where all software is available, including IMOD, Relion, Dynamo, motioncorr2, Gctf, Tomo3d, Aretomo, and NovaCTF. Users can compare different methods for solving the same task instead of wasting time installing software. However, ScipionTomo does not solve the problem of different input/output file types between different software. Furthermore, another new platform can be developed for converting files from different software to uniform formats.

Mathematically, the reconstruction process from a two-dimensional tilt series to a three-dimensional electron density map involves various inverse problems. Specifically, these inverse problems are included in the standard processing discussed above: beam-induced motion fitting, parameter determination of CTF, tilt-series acquisition, noise modeling, and multi-view geometry of three-dimensional reconstruction. In addition, the inherent problems of cryo-ET are the limiting electron dose, the algorithmic enhancement, and the non-unique and unstable numerical solution due to the missing wedge. Thus, a bundle of inverse problems could lead to multiple paths in the pursuit of highly accurate resolution of cells *in situ*. In general, there should be a wide range of mathematical applications in cryo-ET, which may continue to produce outstanding data processing strategies and methods.

With the rapid development of artificial intelligence (AI), many new deep learning-based methods have been designed for cryo-ET like U-net, Resnet, manifold learning, active learning, automatic encoder, and CNN. However, there are several problems. One is that the disunity of training and testing datasets makes the results of comparison among approaches of the same task less reliable. It also confuses users in choosing a suitable one when they want to analyze a new specimen. In addition, input–output gaps also exist with existing software, making some methods less usable. There is much room for improvement in accuracy and speed of calculation. Furthermore, it is certain that more deep learning methods will emerge for 3D reconstruction in cryo-ET. Many new artificial intelligence approaches may be further revised to 3D reconstruction cryo-ET such as methods based on a transformer, manifold learning, and representation theory. Thus, it is necessary to establish a uniform dataset, evaluation criteria, and document format requirements for each task. Cryo-ET will play an increasingly important role in biological science, and may be applied further to medicine, pharmacy, and other fields to promote human progress and the development of science.

Above all, popular deep learning methods and classical mathematical methods have improved the resolution of cryo-ET reconstruction. However, as far as we know, few methods are designed for cryo-ET reconstruction by combining mathematical theory and AI algorithms to complement each other. These kinds of approaches have been used for natural image processing (Wang et al., 2020; Zhao et al., 2021; Li et al., 2022b). The MoAMN method (Zhao et al., 2021) combined the EM algorithm and a denoiser for Gaussian noise to remove a mixture of additive and multiplicative noise. A learnable regularizer provides a good image prior to denoising images in Li et al. (2022b) by integrating the variational method into the architecture of denoiser. Similarly, an image segmentation model with adaptive similarity was developed in Wang et al. (2020). Mathematical knowledge can increase the prior knowledge of AI methods and enrich their interpretability. Meanwhile, the AI approaches improve the mathematical methods to be more flexible and fit to actual data. Therefore, combining mathematical theory and AI algorithms can be an alternative development trend for cryo-ET in the future.

AI professionals and mathematicians have devoted themselves to developing methods for cryo-ET reconstruction procedures. There is still much room to develop new ways to improve resolution besides deep learning methods and mathematical methods (as mentioned above). The combination of the two, and even the integration of biology, physics, and other fields of knowledge. We hope that this review will attract more interdisciplinary researchers to join in developing cryo-ET approaches. In particular, experts from different fields can collaborate to take full advantage of their strength.

## Author contributions

This work is organized by XG. This manuscript is the collaboration of all the authors who are responsible for reading literature, reporting, and discussing the results. The Abstract and Sections 1, 3, 5, 6, and 8–12 are written by CZ. Sections 2, 4, and 7 are written by DL. Details, such as checking language and making images, are completed by CZ. All authors contributed to the article and approved the submitted version.
